# Numerical methods for static shallow shells lying over an obstacle

**DOI:** 10.1007/s11075-019-00830-7

**Published:** 2020-01-10

**Authors:** Paolo Piersanti, Xiaoqin Shen

**Affiliations:** 1 Institute of Mathematics and Scientific Computing, Karl-Franzens-Universität Graz, Heinrichstraße 36, A8010 Graz, Austria; 2 Department of Mathematics, School of Sciences, Xi’an University of Technology, P.O. Box 1243, Yanxiang Road No. 58, Xi’an, 710054 Shaanxi Province China

**Keywords:** Shallow shell, Enriching operator, Nonconforming finite element method, Obstacle problems, Elliptic variational inequalities

## Abstract

In this paper, a finite element analysis to approximate the solution of an obstacle problem for a static shallow shell confined in a half space is presented. To begin with, we establish, by relying on the properties of enriching operators, an estimate for the approximate bilinear form associated with the problem under consideration. Then, we conduct an error analysis and we prove the convergence of the proposed numerical scheme.

## Introduction

The study of unilateral contact problems in elasticity arises in many applicative fields such as structural mechanics and civil engineering. Obstacle problems have lately been studied in, for instance, [20, 21, 23, 24, 36, 40].

The numerical analysis of obstacle problems has been arising the interest of many scientists since the late 1990s. In this direction, a very direct and mathematically elegant approach is the one making use of enriching operators, the properties of which were studied by S. C. Brenner and her collaborators in the seminal papers [1–3, 5]. These general theoretical results were then used to study finite element methods for obstacle problems, which can be found in [7] and [6]. Nonconforming finite element methods for obstacle problem were also studied in [11].

In this paper, we study the displacement of a static shallow shell lying over a planar obstacle from the numerical point of view, using a suitable finite element method. Shallow shells theory, which is extensively described, for instance, in the books [16] and [44], is widely used in engineering (see, e.g., the papers [31, 41–43, 46]). According to this theory, the problem under examination is modelled in terms of a fourth-order differential operator (cf., e.g., [16]). The theory of finite element methods for fourth-order problems governed by variational inequalities has been investigated, for instance, in the references [8, 25, 30, 32].

Our mathematical model of an obstacle problem for a linearly elastic shallow shell in the static case is inspired by that of Léger and Miara (cf. [34] and [35]). To our best knowledge, there is no reference on the study of numerical analysis of obstacle problems for linearly elastic shallow shells.

In this paper, we extend the method proposed in [7] and [6] to derive error estimates for the solution to the obstacle problem under for linearly elastic shallow shells under consideration. The fact that the unknown is a vector field is the main difficulty to cope with in order for proving that the residual of the difference between the exact solution and the approximate solution approaches zero as the mesh size approaches zero.

In order to derive the sought convergence, it was necessary to improve and generalize a number of preparatory results for enriching operators (cf., e.g., [1–3, 5]), and another number of preparatory results related to the convergence analysis of the scheme (cf., e.g., [7]).

The paper is divided into five sections (including this one). In Section 2, we present some background and notation. In Section 3, we establish some properties of the enriching operator associated with the variational formulation of the problem under consideration and an estimate for Morley’s triangle, used to approximate the transverse component of the displacement. In Section 4, following [7] and [6], we introduce an intermediary problem and we prove some technical preparatory lemmas. Finally, in Section 5, the error estimate is derived as a result of an application of the previous results.

## Background and notation

For an overview about the classical notions of differential geometry used in this paper, see, e.g., [17] or [18] while, for an overview about the classical notions of functional analysis used in this paper, see, e.g., [19]. Latin indices, except *h*, take their values in the set {1,2,3} while Greek indices, except *ν* and *ε*, take their values in the set {1,2}. The notation *δ*_*αβ*_ designates the Kronecker symbol. Given an open subset *Ω* of $\mathbb {R}^{n}$, where *n* ≥ 1, we denote the usual Lebesgue and Sobolev spaces by *L*^2^(*Ω*), *H*^1^(*Ω*), ${H^{1}_{0}} (\varOmega )$, *H*^2^(*Ω*), or ${H^{2}_{0}}(\varOmega )$; the notation $\mathcal {D} (\varOmega )$ designates the space of all functions that are infinitely differentiable over *Ω* and have compact supports in *Ω*; the notation $\mathbb {P}_{k}(\varOmega )$ designates the space of all polynomials of degree ≤ *k* defined over *Ω*; and the notation $\mathbb {P}_{k}$ designates the space of all polynomials of degree ≤ *k* defined over $\mathbb {R}^{n}$. The Euclidean norm of any point *x* ∈*Ω* is denoted by |*x*|. In what follows, the compact notation ∥⋅∥_*m*,*p*,*Ω*_, where *m* ≥ 1 is an integer and *p* ≥ 1, designates the norm of the Sobolev space *W*^*m*,*p*^(*Ω*). The special notation ∥⋅∥_*m*,*Ω*_, where *m* ≥ 1 is an integer, denotes the norm of the space *H*^*m*^(*Ω*). If *m* = 0, then

$$ \|\cdot\|_{0,\varOmega}:=\|\cdot\|_{L^{2}(\varOmega)} $$

and, more generically,

$$ \|\cdot\|_{0,p,\varOmega}:=\|\cdot\|_{L^{p}(\varOmega)} \quad\text{ for all } p\ge 1. $$

The special notation |⋅|_*m*,*Ω*_, where *m* ≥ 1 is an integer, denotes the standard semi-norm of the space *H*^*m*^(*Ω*).

Let $\omega \subset \mathbb {R}^{2}$ be a convex polygonal domain, namely a non-empty bounded open connected subset of $\mathbb {R}^{2}$ with Lipschitz continuous boundary *γ* := *∂ω* and such that *ω* is all *on the same side of γ*. Let *y* = (*y*_*α*_) denote a generic point in $\overline {\omega }$ and let *∂*_*α*_ := *∂*/*∂y*_*α*_ and *∂*_*αβ*_ := *∂*^2^/*∂y*_*α*_*∂y*_*β*_.

Referring to [22] (see also Section 3.1 of [16] and see also [44]), we recall the rigorous definition of a linearly elastic shallow shell (from now on *shallow shell*). We assume that for each *ε* > 0, we are given a function $\theta ^{\varepsilon } \in \mathcal {C}^{3}(\overline {\omega })$. We can then define the middle surface of the corresponding shallow shell having thinness equal to 2*ε* as follows:

$$ \hat{\omega}^{\varepsilon}:=\{(y, \theta^{\varepsilon}(y));y\in \overline{\omega}\}. $$

A rigorous criterion for defining a shallow shell is provided by the *existence* of a function $\theta \in \mathcal {C}^{3}(\overline {\omega })$, independent of *ε*, such that

1 $$  \theta^{\varepsilon}(y)=\varepsilon \theta(y) \text{ for all }y \in \overline{\omega}. $$

This means that, up to an additive constant, the mapping $\theta ^{\varepsilon }:\overline {\omega } \to \mathbb {R}$, measuring the deviation of the middle surface of the reference configuration of the shell from a plane, should be of the same order as the thinness of the shell. The shallow shells here considered are made of a homogeneous and isotropic material, they are clamped on their lateral boundary, and they are subjected to both applied body forces and applied surface forces. The elastic behavior of the shallow shell is then described by means of its two Lamé constants *λ* ≥ 0 and *μ* > 0 (cf., e.g., [15]).

In what follows, *ν* denotes the outer unit normal vector field to the boundary *γ* and *∂*_*ν*_ denotes the outer unit normal derivative operator along *γ*.

The function space over which the problem is posed is the following:

$$ \boldsymbol{V}(\omega):=\{\boldsymbol{\eta}=(\eta_{i}) \in H^{1}(\omega)\times H^{1}(\omega)\times H^{2}(\omega); \eta_{i}=\partial_{\nu}\eta_{3}=0 \text{ on }\gamma\}. $$

We equip the space ***V***(*ω*) with the norm ∥⋅∥_***V***(*ω*)_ defined as follows:

$$ \|\boldsymbol{\xi}\|_{\boldsymbol{V}(\omega)}:=\|\xi_{1}\|_{1,\omega}+\|\xi_{2}\|_{1,\omega}+\|\xi_{3}\|_{2,\omega} \quad\text{ for all }\boldsymbol{\xi} \in \boldsymbol{V}(\omega). $$

The corresponding semi-norm |⋅|_***V***(*ω*)_ is defined by

$$ |\boldsymbol{\eta}|_{\boldsymbol{V}(\omega)}:=|\eta_{1}|_{1,\omega}+|\eta_{2}|_{1,\omega}+|\eta_{3}|_{2,\omega} \quad\text{ for all }\boldsymbol{\eta} \in \boldsymbol{V}(\omega). $$

The obstacle problem studied in [34] and [35] is modelled by a set of variational equations and a set of variational inequalities and, besides, its solution is a *Kirchhoff-Love field* (see, for instance, Section 3.4 of [16]). As a result, we can “separate” the transverse component of the displacement vector field from the tangential components of the displacement vector field. We thus define the space associated with the tangential components by

$$ \boldsymbol{V}_{H}(\omega):=\{\boldsymbol{\eta}_{H}=(\eta_{\alpha}) \in H^{1}(\omega)\times H^{1}(\omega); \eta_{\alpha}=0 \text{ on }\gamma\}, $$

and the space associated with the transverse component by

$$ V_{3}(\omega):={H^{2}_{0}}(\omega). $$

Observe that

$$ \boldsymbol{V}(\omega)=\boldsymbol{V}_{H}(\omega) \times V_{3}(\omega). $$

The “physical” obstacle is here represented by the plane *x*_3_ = 0 and, in what follows, we assume that *𝜃* > 0 in $\overline {\omega }$. This implies *𝜃*^*ε*^ > 0 in $\overline {\omega }$, i.e., the middle surface of the considered shallow shell is assumed to be *above* the obstacle and *not in contact with the obstacle*.

In what follows, we state the scaled two-dimensional limit problem, which slightly differs from the one obtained in [34] and [35] as a result of a rigorous asymptotic analysis, where only the transverse component of the displacement is subjected to the geometrical constraint associated with the obstacle. More specifically, the transverse component of the displacement field belongs to the following non-empty, closed, and convex set of the space *V*_3_(*ω*) (see [34]):

2 $$  K_{3}(\omega):=\{\eta_{3} \in V_{3}(\omega); \theta+\eta_{3} \ge 0 \text{ almost everywhere in }\omega\}. $$

By virtue of the Rellich-Kondrachov theorem, the compact embedding $H^{2}(\omega ) \hookrightarrow \hookrightarrow \mathcal {C}^{0}(\overline {\omega })$ holds (the symbol “↪↪” denotes a compact embedding and the space $\mathcal {C}^{0}(\overline {\omega })$ is equipped with the $\sup $-norm). Hence, by virtue of the fact that $\theta \in \mathcal {C}^{3}(\overline {\omega })$, the set *K*_3_(*ω*) defined in (2) also takes the following form:

3 $$  K_{3}(\omega)=\{\eta_{3} \in V_{3}(\omega); \theta+\eta_{3} \ge 0 \text{ in }\overline{\omega}\}. $$

Let

$$ \varOmega := \omega \times \left] - 1, 1 \right[ , $$

and let *x* = (*x*_*i*_) denote a generic point in the set $\overline {\varOmega }$. With each point $x = (x_{i}) \in \overline {\varOmega }$, we associate the point $x^{\varepsilon } = (x^{\varepsilon }_{i})$ defined by

$$ x^{\varepsilon}_{\alpha} := x_{\alpha} = y_{\alpha} \text{ and } x^{\varepsilon}_{3} := \varepsilon x_{3}, $$

so that $\partial ^{\varepsilon }_{\alpha } = \partial _{\alpha }$ and $\partial ^{\varepsilon }_{3} = \displaystyle \frac {1}{\varepsilon } \partial _{3}$.

We *assume* that the shallow shell under consideration is subjected to *applied body forces* whose density per unit volume is defined by means of its covariant components $f^{\varepsilon }_{i} \in L^{2}(\omega \times (-\varepsilon ,\varepsilon ))$ and *applied surface forces* whose density per unit area is defined by means of its covariant components $g^{+,\varepsilon }_{i} \in L^{2}(\omega \times \{\varepsilon \})$. Applied surface forces associated with the lower face of the reference configuration of the shallow shell are not to be considered since the obstacle is assumed to be rigid.

We also *assume* that there exist functions *f*_*i*_ ∈ *L*^2^(*Ω*) and $g_{i}^{+} \in L^{2}(\omega \times \{1\})$*independent of* *ε* such that the following *assumptions on the data* hold:

$$ \begin{array}{@{}rcl@{}} f^{\varepsilon}_{\alpha}(x^{\varepsilon})&=&\varepsilon^{2} f_{\alpha}(x)\text{ at each }x=(x_{i}) \in \varOmega,\\ f^{\varepsilon}_{3}(x^{\varepsilon})&=&\varepsilon^{3} f_{3}(x)\text{ at each }x=(x_{i}) \in \varOmega,\\ g^{+,\varepsilon}_{\alpha}(x^{\varepsilon})&=&\varepsilon^{3} g_{\alpha}^{+}(x)\text{ at each }x=(x_{i}) \in \omega \times \{1\},\\ g^{+,\varepsilon}_{3}(x^{\varepsilon})&=&\varepsilon^{4} g_{3}^{+}(x)\text{ at each }x=(x_{i}) \in \omega \times \{1\}. \end{array} $$

We are now ready to state the *scaled* limit problem $\mathcal {P}(\omega )$, which slightly differs from the one found in [34] and [35].

### **Problem 1**

$\mathcal {P}(\omega )$ Find ***ζ*** = (***ζ***_*H*_,*ζ*_3_) ∈***V***_*H*_(*ω*) × *K*_3_(*ω*) satisfying the following variational inequalities

$$ \begin{array}{@{}rcl@{}} &&-{\int}_{\omega}m_{\alpha\beta}(\zeta_{3})\partial_{\alpha\beta}(\eta_{3}-\zeta_{3}) \mathrm{d} y + {\int}_{\omega} n_{\alpha\beta}^{\theta}(\boldsymbol{\zeta}) \partial_{\alpha} \theta \partial_{\beta}(\eta_{3}-\zeta_{3}) \mathrm{d} y\\ &&\ge {\int}_{\omega} p_{3} (\eta_{3}-\zeta_{3}) \mathrm{d} y - {\int}_{\omega} s_{\alpha} \partial_{\alpha}(\eta_{3}-\zeta_{3})\mathrm{d} y\\ &&\text{ for all } \eta_{3} \in K_{3}(\omega), \end{array} $$

and the following variational equations

$$ {\int}_{\omega} n_{\alpha\beta}^{\theta}(\boldsymbol{\zeta})\partial_{\beta} \eta_{\alpha} \mathrm{d} y={\int}_{\omega} p_{\alpha} \eta_{\alpha} \mathrm{d} y \quad\text{ for all }\boldsymbol{\eta}_{H}=(\eta_{\alpha}) \in \boldsymbol{V}_{H}(\omega), $$

where

$$ \left\{\begin{array}{lll} &\lambda\ge 0, \mu>0\quad\text{ are the Lam\'{e} constants},\\ &m_{\alpha\beta}(\zeta_{3}):=-\frac{4\lambda\mu}{3(\lambda+2\mu)} {\Delta} \zeta_{3} \delta_{\alpha\beta}-\frac{4}{3}\mu \partial_{\alpha\beta} \zeta_{3},\\ &e_{\alpha\beta}^{\theta}(\boldsymbol{\zeta}):=\frac{1}{2}(\partial_{\alpha} \zeta_{\beta}+\partial_{\beta}\zeta_{\alpha})+\frac{1}{2}(\partial_{\alpha}\theta\partial_{\beta}\zeta_{3}+\partial_{\beta}\theta\partial_{\alpha}\zeta_{3}),\\ &n_{\alpha\beta}^{\theta}(\boldsymbol{\zeta}):=\frac{4\lambda\mu}{\lambda+2\mu}e_{\sigma\sigma}^{\theta}(\boldsymbol{\zeta})\delta_{\alpha\beta}+4\mu e_{\alpha\beta}^{\theta}(\boldsymbol{\zeta}),\\ &p_{i}:={\int}_{-1}^{1}f_{i} \mathrm{d} x_{3}+g_{i}^{+},\\ &s_{\alpha}:={\int}_{-1}^{1} x_{3} f_{\alpha} \mathrm{d} x_{3}+g_{\alpha}^{+}. \end{array}\right. $$

$\blacksquare $

Likewise, since $\theta ^{\varepsilon } \in \mathcal {C}^{3}(\overline {\omega })$, we define the non-empty closed convex set $K_{3}^{\varepsilon }(\omega )$ by

4 $$  K_{3}^{\varepsilon}(\omega):=\{\eta_{3} \in V_{3}(\omega); \theta^{\varepsilon}+\eta_{3} \ge 0 \text{ in }\overline{\omega}\}. $$

The next step consists in *de-scaling* Problem $\mathcal {P}(\omega )$. More specifically, the solution (***ζ***_*H*_,*ζ*_3_) is *de-scaled* as follows (cf. [16])

$$ \begin{array}{@{}rcl@{}} \boldsymbol{\zeta}_{H}^{\varepsilon}=\varepsilon^{2} \boldsymbol{\zeta}_{H} &\quad\text{ in }\omega,\\ \zeta_{3}^{\varepsilon}=\varepsilon \zeta_{3} &\quad\text{ in }\omega. \end{array} $$

Thanks to (1), if *ζ*_3_ ∈ *K*_3_(*ω*), then $\zeta _{3}^{\varepsilon } \in K_{3}^{\varepsilon }(\omega )$. The *de-scaled* problem $\mathcal {P}^{\varepsilon }(\omega )$ can be thus stated and constitutes the point of departure of our numerical analysis.

### **Problem 2**

$\mathcal {P}^{\varepsilon }(\omega )$ Find $\boldsymbol {\zeta }^{\varepsilon }=(\boldsymbol {\zeta }_{H}^{\varepsilon }, \zeta _{3}^{\varepsilon }) \in \boldsymbol {V}_{H}(\omega ) \times K_{3}^{\varepsilon }(\omega )$ satisfying the following variational inequalities:

5 $$ \begin{array}{@{}rcl@{}} &&-{\int}_{\omega}m_{\alpha\beta}^{\varepsilon}(\zeta_{3}^{\varepsilon})\partial_{\alpha\beta}(\eta_{3}-\zeta_{3}^{\varepsilon}) \mathrm{d} y + {\int}_{\omega} n_{\alpha\beta}^{\theta,\varepsilon}(\boldsymbol{\zeta}^{\varepsilon}) (\partial_{\alpha} \theta^{\varepsilon}) \partial_{\beta}(\eta_{3}-\zeta_{3}^{\varepsilon}) \mathrm{d} y\\ &&\ge {\int}_{\omega} p_{3}^{\varepsilon} (\eta_{3}-\zeta_{3}^{\varepsilon}) \mathrm{d} y - {\int}_{\omega} s_{\alpha}^{\varepsilon} \partial_{\alpha}(\eta_{3}-\zeta_{3}^{\varepsilon})\mathrm{d} y\\ &&\text{ for all }\eta_{3} \in K_{3}^{\varepsilon}(\omega), \end{array} $$

and the following variational equations:

6 $$  {\int}_{\omega} n_{\alpha\beta}^{\theta,\varepsilon}(\boldsymbol{\zeta}^{\varepsilon})\partial_{\beta} \eta_{\alpha} \mathrm{d} y={\int}_{\omega} p_{\alpha}^{\varepsilon} \eta_{\alpha} \mathrm{d} y \quad \text{ for all }\boldsymbol{\eta}_{H}=(\eta_{\alpha}) \in \boldsymbol{V}_{H}(\omega), $$

where

7 $$  \left\{\begin{array}{llll} &\lambda\ge 0, \mu>0\quad\text{ are the Lam\'{e} constants},\\ &m_{\alpha\beta}^{\varepsilon}(\zeta_{3}^{\varepsilon}):=-\varepsilon^{3}\left\{\frac{4\lambda\mu}{3(\lambda+2\mu)} {\Delta} \zeta_{3}^{\varepsilon} \delta_{\alpha\beta}+\frac{4}{3}\mu \partial_{\alpha\beta} \zeta_{3}^{\varepsilon}\right\},\\ &e_{\alpha\beta}^{\theta,\varepsilon}(\boldsymbol{\zeta}^{\varepsilon}):=\frac{1}{2}(\partial_{\alpha} \zeta_{\beta}^{\varepsilon}+\partial_{\beta}\zeta_{\alpha}^{\varepsilon})+\frac{1}{2}(\partial_{\alpha}\theta^{\varepsilon} \partial_{\beta}\zeta_{3}^{\varepsilon}+\partial_{\beta}\theta^{\varepsilon} \partial_{\alpha}\zeta_{3}^{\varepsilon}),\\ &n_{\alpha\beta}^{\theta,\varepsilon}(\boldsymbol{\zeta}^{\varepsilon}):=\varepsilon\left\{\frac{4\lambda\mu}{\lambda+2\mu}e_{\sigma\sigma}^{\theta,\varepsilon}(\boldsymbol{\zeta}^{\varepsilon})\delta_{\alpha\beta}+4\mu e_{\alpha\beta}^{\theta,\varepsilon}(\boldsymbol{\zeta}^{\varepsilon})\right\},\\ &p_{i}^{\varepsilon}:={\int}_{-\varepsilon}^{\varepsilon}f_{i}^{\varepsilon} \mathrm{d} x_{3}^{\varepsilon}+g_{i}^{+,\varepsilon},\\ &s_{\alpha}^{\varepsilon}:={\int}_{-\varepsilon}^{\varepsilon} x_{3}^{\varepsilon} f_{\alpha}^{\varepsilon} \mathrm{d} x_{3}^{\varepsilon}+ \varepsilon g_{\alpha}^{+,\varepsilon}. \end{array}\right. $$

$\blacksquare $

Clearly, (5) and (6) can be combined into a single system of variational equations, whose left-hand side is associated with the symmetric bilinear form *b*(⋅,⋅) given by (cf. Sections 3.5, 3.6 and 3.7 of [16])

8 $$  \begin{array}{llll} b(\boldsymbol{\zeta}^{\varepsilon},\boldsymbol{\eta})&=-{\int}_{\omega} m_{\alpha\beta}^{\varepsilon}(\zeta_{3}^{\varepsilon}) \partial_{\alpha\beta} \eta_{3} \mathrm{d} y + {\int}_{\omega} n_{\alpha\beta}^{\theta,\varepsilon}(\boldsymbol{\zeta}^{\varepsilon}) (\partial_{\alpha} \theta^{\varepsilon}) \partial_{\beta}\eta_{3} \mathrm{d} y\\ &\quad +{\int}_{\omega} n_{\alpha\beta}^{\theta,\varepsilon}(\boldsymbol{\zeta}^{\varepsilon})\partial_{\beta} \eta_{\alpha} \mathrm{d} y. \end{array} $$

A straightforward computation shows that

$$ \begin{array}{llll} b(\boldsymbol{\eta},\boldsymbol{\eta})&:=\displaystyle{\int}_{\omega}\frac{4\lambda\mu}{\lambda+2\mu}\left\{\frac{\varepsilon^{3}}{3}({\Delta} \eta_{3})^{2}+\varepsilon (e_{\sigma\sigma}^{\theta,\varepsilon}(\boldsymbol{\eta}))^{2}\right\} \mathrm{d} y\\ &\quad+4\mu \left\{\frac{\varepsilon^{3}}{3}\sum\limits_{\alpha, \beta}\|\partial_{\alpha\beta}\eta_{3}\|_{0,\omega}^{2}+\varepsilon\sum\limits_{\alpha, \beta} \|e_{\alpha\beta}^{\theta,\varepsilon}(\boldsymbol{\eta})\|_{0,\omega}^{2}\right\}, \end{array} $$

for all ***η*** ∈***V***(*ω*).

Likewise, we associate the sum of the right-hand sides of (5) and (6) with a linear and continuous form *ℓ* defined as follows:

9 $$  \ell(\boldsymbol{\eta}):={\int}_{\omega} p_{i}^{\varepsilon} \eta_{i} \mathrm{d} y - {\int}_{\omega} s_{\alpha}^{\varepsilon} \partial_{\alpha}\eta_{3} \mathrm{d} y \quad\text{ for all } \boldsymbol{\eta}\in \boldsymbol{V}(\omega). $$

The energy functional associated with the variational formulation in Problem $\mathcal {P}^{\varepsilon }(\omega )$ takes the following form:

$$ J^{\varepsilon}(\boldsymbol{\eta})=\frac{1}{2} b(\boldsymbol{\eta},\boldsymbol{\eta}) -\ell(\boldsymbol{\eta}), \quad \text{ for all } \boldsymbol{\eta} \in \boldsymbol{V}_{H}(\omega) \times K_{3}^{\varepsilon}(\omega). $$

As a result, Problem $\mathcal {P}^{\varepsilon }(\omega )$ is equivalent to finding $\boldsymbol {\zeta }^{\varepsilon }=(\boldsymbol {\zeta }_{H}^{\varepsilon }, \zeta _{3}^{\varepsilon }) \in $$ \boldsymbol {V}_{H}(\omega ) \times K_{3}^{\varepsilon }(\omega )$ such that

$$ J^{\varepsilon}(\boldsymbol{\zeta}^{\varepsilon})=\min\{J^{\varepsilon}(\boldsymbol{\eta});\boldsymbol{\eta} \in \boldsymbol{V}_{H}(\omega) \times K_{3}^{\varepsilon}(\omega)\}. $$

The bilinear form *b*(⋅,⋅) is continuous, i.e., there exists a constant *M* > 0 such that

$$ b(\boldsymbol{\xi}, \boldsymbol{\eta}) \le M \|\boldsymbol{\xi}\|_{\boldsymbol{V}(\omega)} \|\boldsymbol{\eta}\|_{\boldsymbol{V}(\omega)} \quad\text{ for all }\boldsymbol{\xi}, \boldsymbol{\eta} \in \boldsymbol{V}(\omega). $$

By Theorem 3.6-1 of [16], such a bilinear form *b*(⋅,⋅) is ***V***(*ω*)-elliptic, i.e., there exists a constant *α* > 0 such that

$$ b(\boldsymbol{\eta}, \boldsymbol{\eta}) \ge \alpha \|\boldsymbol{\eta}\|_{\boldsymbol{V}(\omega)}^{2} \quad\text{ for all }\boldsymbol{\eta}\in \boldsymbol{V}(\omega). $$

As a result, Problem $\mathcal {P}^{\varepsilon }(\omega )$ admits a unique solution $\boldsymbol {\zeta }^{\varepsilon }=(\boldsymbol {\zeta }^{\varepsilon }_{H}, \zeta ^{\varepsilon }_{3})$ which belongs to $\boldsymbol {V}_{H}(\omega )\times K_{3}^{\varepsilon }(\omega )$ and satisfying

10 $$  b(\boldsymbol{\zeta}^{\varepsilon},\boldsymbol{\eta}-\boldsymbol{\zeta}^{\varepsilon})\ge\ell(\boldsymbol{\eta}-\boldsymbol{\zeta}^{\varepsilon})\quad\text{ for all }\boldsymbol{\eta}=(\boldsymbol{\eta}_{H}, \eta_{3})\in \boldsymbol{V}_{H}(\omega)\times K_{3}^{\varepsilon}(\omega) $$

or, equivalently, there exists a unique $\boldsymbol {\zeta }^{\varepsilon }=(\boldsymbol {\zeta }_{H}^{\varepsilon }, \zeta _{3}^{\varepsilon }) \in \boldsymbol {V}_{H}(\omega ) \times K_{3}^{\varepsilon }(\omega )$ such that

$$ J^{\varepsilon}(\boldsymbol{\zeta}^{\varepsilon})=\min\{J^{\varepsilon}(\boldsymbol{\eta});\boldsymbol{\eta} \in \boldsymbol{V}_{H}(\omega) \times K_{3}^{\varepsilon}(\omega)\}. $$

## A finite element method for the obstacle problem

In this section, we present a suitable finite element method to approximate the solution to Problem $\mathcal {P}^{\varepsilon }(\omega )$. Following [14] and [4] (see also [12], [13], [29], and [37]), we recall some basic terminology and definitions. In what follows, the letter *h* denotes a quantity approaching zero. For brevity, the same notation *C* (with or without subscripts) designates a positive constant independent of *h*, which can take different values at different places. We denote by $(\mathcal {T}_{h})_{h>0}$ a *family of triangulations of the polygonal domain* $\overline {\omega }$ made of triangles and we let *T* denote any element of such a family. Let us first recall, following [14] and [4], the *rigorous* definition of *finite element* in $\mathbb {R}^{n}$, where *n* ≥ 1 is an integer. A *finite element* in $\mathbb {R}^{n}$ is a *triple* $(T,P, \mathcal {N})$ where:

(i) *T* is a closed subset of $\mathbb {R}^{n}$ with non-empty interior and Lipschitz continuous boundary,
(ii) *P* is a finite dimensional space of real-valued functions defined over *T*,
(iii) $\mathcal {N}$ is is a finite set of linearly independent linear forms *N*_*i*_, $1 \le i \le \dim P$, defined over the space *P*.

By definition, it is assumed that the set $\mathcal {N}$ is *P-unisolvent* in the following sense: given any real scalars *α*_*i*_, $1\le i \le \dim P$, there exists a unique function *g* ∈ *P* which satisfies

$$ N_{i}(g)=\alpha_{i}, \quad 1 \le i \le \dim P. $$

It is henceforth assumed that the *degrees of freedom*, *N*_*i*_, lie in the dual space of a function space larger than *P* like, for instance, a Sobolev space (see [4]). For brevity, we shall conform our terminology to the one of [14], calling the sole set *T* a finite element. Define the *diameter* of any finite element *T* as follows:

$$ h_{T}=\text{diam }T:= \max\limits_{x,y \in T} |x-y|. $$

Let us also define

$$ \rho_{T}:=\sup\{\text{diam }B; B \text{ is a ball contained in }T\}. $$

A triangulation $\mathcal {T}_{h}$ is said to be *regular* (cf., e.g., [14]) if:

(i) There exists a constant *σ* > 0, independent of *h*, such that
  $$ \text{for all }T \in \mathcal{T}_{h},\quad \frac{h_{T}}{\rho_{T}} \le \sigma. $$
(ii) The quantity $h:=\max \limits \{h_{T}>0; T \in \mathcal {T}_{h}\} $ approaches zero.

A triangulation $\mathcal {T}_{h}$ is said to satisfy *an inverse assumption* (cf., e.g., [14]) if there exists a constant *κ* > 0 such that

$$ \text{for all }~T \in \mathcal{T}_{h},\quad \frac{h}{h_{T}} \le \kappa. $$

There is of course an ambiguity in the meaning of *h*, which was first regarded as a parameter associated with the considered family of triangulations, and which next denotes a geometrical entity. Nevertheless, in this paper, we have conformed to this standard notation (see [14]). In the rest of this section, the *parameter* *h* is assumed to be fixed and we also assume that the triangulation $\mathcal {T}_{h}$ under consideration is regular and satisfies the aforementioned inverse assumption. Let $\mathcal {V}_{h}$ be the set of *all* of the *nodal points* of $\mathcal {T}_{h}$, let *p* denote any point of $\mathcal {V}_{h}$ and let $\mathcal {E}_{h}$ be the set of *open edges* of $\mathcal {T}_{h}$, in the sense that

$$ \text{any edge }e \in \mathcal{E}_{h} \text{ is isomorphic to the open interval }(0,1). $$

The forthcoming finite element analysis will be carried out using triangles of type (1) (see Figure 2.2.1 of [14]) to approximate the tangential components of the displacement vector field and Morley’s triangles (see [39] and also [14]) to approximate the transverse component of the displacement vector field. In this case, the set $\mathcal {V}_{h}$ consists of all the vertices and all the midpoints of the triangulation $\mathcal {T}_{h}$. Let *V*_1,*h*_ and *V*_2,*h*_ be two finite dimensional spaces such that *V*_1,*h*_ × *V*_2,*h*_ ⊂***V***_*H*_(*ω*) and let (see, e.g., [14] and [6])

$$ \begin{array}{@{}rcl@{}} V_{3,h}&:=\{\eta \in L^{2}(\omega); \eta_{T} \in \mathbb{P}_{2}(T), \eta \text{ is continuous at the vertices},\\ &\qquad \partial_{\nu} \eta \text{ continuous at the midpoint of the edges} \} \end{array} $$

be the finite dimensional space associated with Morley’s triangle. Define

$$ \boldsymbol{V}_{h}:=V_{1,h} \times V_{2,h}\times V_{3,h}. $$

We henceforth denote by *η*_*T*_ the restriction of any function *η* ∈ *L*^2^(*ω*) to the finite element *T*. We denote by $\tilde {V}_{3,h}$ the subspace of *V*_3,*h*_ for which *η*(*a*_*k*_) = 0, for all the vertices *a*_*k*_ ∈ *γ* and *∂*_*ν*_*η*(*b*_*k*_) = 0, for all the edges midpoints *b*_*k*_ such that *b*_*k*_ ∈ *γ*. Define the space

$$ \tilde{\boldsymbol{V}}_{h}:= V_{1,h}\times V_{2,h}\times \tilde{V}_{3,h}. $$

Since $\tilde {V}_{3,h}$ is not contained in $\mathcal {C}^{0}(\overline {\omega })$ (see, e.g., [33] and [29]), we have

$$ \tilde{V}_{3,h} \not\subset V_{3}(\omega). $$

Define the space

$$ H^{2}(\omega,\mathcal{T}_{h}):=\{\eta\in L^{2}(\omega);\eta_{T} \in H^{2}(T) \text{ for all }T \in \mathcal{T}_{h} \} $$

and introduce the semi-norm

$$ \eta \in H^{2}(\omega,\mathcal{T}_{h}) \mapsto \|\eta\|_{h}:=\left( \sum\limits_{T \in \mathcal{T}_{h}}|\eta|_{2,T}^{2}\right)^{1/2}, $$

which becomes a norm over the space $\tilde {V}_{3,h}$ (cf. [6]). As a result, the mapping

$$ \boldsymbol{\eta}_{h} \in \tilde{\boldsymbol{V}}_{h} \mapsto \|\boldsymbol{\eta}_{h}\|:=\|\eta_{1,h}\|_{1,\omega}+\|\eta_{2,h}\|_{1,\omega}+\|\eta_{3,h}\|_{h}, $$

is a norm over the space $\tilde {\boldsymbol {V}}_{h}$.

Define the space

$$ V_{3}(\omega)+\tilde{V}_{3,h}:=\{\xi_{3}=\eta_{3}+\eta_{3,h}; \eta_{3} \in V_{3}(\omega) \text{ and }\eta_{3,h} \in \tilde{V}_{3,h}\}. $$

Following [7] and [14], we define the approximate bilinear form *b*_*h*_(⋅,⋅), associated with the bilinear form *b* defined in (8), as follows:

$$ b_{h}: \left( \boldsymbol{V}_{H}(\omega) \times (V_{3}(\omega)+\tilde{V}_{3,h})\right) \times \left( \boldsymbol{V}_{H}(\omega) \times (V_{3}(\omega)+\tilde{V}_{3,h})\right) \to \mathbb{R} $$

is such that *b*_*h*_|_***V***(*ω*)×***V***(*ω*)_ = *b*, i.e.,

$$ b_{h}(\boldsymbol{\xi},\boldsymbol{\eta})=b(\boldsymbol{\xi},\boldsymbol{\eta})\quad\text{ for all }\boldsymbol{\xi}, \boldsymbol{\eta}\in \boldsymbol{V}(\omega) $$

and such that

$$ \begin{array}{@{}rcl@{}} b_{h}(\boldsymbol{\xi}_{h},\boldsymbol{\eta}_{h})&=&-\sum\limits_{T \in \mathcal{T}_{h}}{\int}_{T} m_{\alpha\beta}^{\varepsilon}(\xi_{3,h}) \partial_{\alpha\beta} \eta_{3,h} \mathrm{d} y + \sum\limits_{T \in \mathcal{T}_{h}} {\int}_{T} n_{\alpha\beta}^{\theta,\varepsilon}(\boldsymbol{\xi}_{h}) (\partial_{\alpha} \theta^{\varepsilon}) \partial_{\beta}\eta_{3,h} \mathrm{d} y\\ &&+{\sum}_{T \in \mathcal{T}_{h}} {\int}_{T} n_{\alpha\beta}^{\theta,\varepsilon}(\boldsymbol{\xi}_{h})\partial_{\beta} \eta_{\alpha,h} \mathrm{d} y,\quad\text{ for all }\boldsymbol{\xi}_{h}, \boldsymbol{\eta}_{h} \in \tilde{\boldsymbol{V}}_{h}. \end{array} $$

Therefore, the bilinear form *b*_*h*_(⋅,⋅) is continuous over $\tilde {\boldsymbol {V}}_{h}$, i.e., there exists *M* > 0, independent of *h*, such that

$$ b_{h}(\boldsymbol{\xi},\boldsymbol{\eta})\le M\|\boldsymbol{\xi}\| \|\boldsymbol{\eta}\|\quad\text{ for all }\boldsymbol{\xi}, \boldsymbol{\eta}\in \tilde{\boldsymbol{V}}_{h}. $$

Besides, in view of Theorem 3.4-1 of [16] and the theory presented in [14], the bilinear form *b*_*h*_(⋅,⋅) is $\tilde {\boldsymbol {V}}_{h}$-elliptic, namely, there exists *α* > 0, independent of *h*, such that

$$ b_{h}(\boldsymbol{\eta},\boldsymbol{\eta}) \ge \alpha \|\boldsymbol{\eta}\|^{2}\quad\text{ for all }\boldsymbol{\eta}\in \tilde{\boldsymbol{V}}_{h}. $$

Let us now define the ***V***_*h*_ interpolation operator $\boldsymbol {\varPi }_{h}:\mathcal {C}^{0}(\overline {\omega }) \times \mathcal {C}^{0}(\overline {\omega }) \times H^{2}(\omega )\to \boldsymbol {V}_{h}$ as follows

$$ \boldsymbol{\varPi}_{h} \boldsymbol{\xi}:= (\varPi_{1,h} \xi_{1}, {\varPi}_{2,h} \xi_{2}, {\varPi}_{3,h} \xi_{3}) \quad\text{ for all }\boldsymbol{\xi}\in \mathcal{C}^{0}(\overline{\omega}) \times \mathcal{C}^{0}(\overline{\omega}) \times H^{2}(\omega), $$

where *π*_*i*,*h*_ is the standard *V*_*i*,*h*_ interpolation operator (cf., e.g., [14] and [4]). It thus results that the interpolation operator **π**_*h*_ satisfies the following properties

$$ \begin{array}{@{}rcl@{}} &&({\varPi}_{j,h} \xi_{j})(p)=\xi_{j}(p)\quad\text{ for all integers }1 \le j \le 3 \text{ and all vertices }p \in \mathcal{V}_{h},\\ &&{\int}_{e} \partial_{\nu_{e}} ({\varPi}_{3,h} \xi_{3}) \mathrm{d} s={\int}_{e}\partial_{\nu_{e}} \xi_{3} \mathrm{d} s\quad\text{ for all }e\in\mathcal{E}_{h}, \end{array} $$

where *ν*_*e*_ is outer unit normal vector to the edge *e*. Define the space

$$ \boldsymbol{H}(\omega):=H^{2}(\omega)\times H^{2}(\omega) \times H^{3}(\omega) $$

and equip it with the norm

$$ \|\boldsymbol{\xi}\|_{\omega}:=\|\xi_{1}\|_{2,\omega}+\|\xi_{2}\|_{2,\omega}+\|\xi_{3}\|_{3,\omega} \quad\text{ for all } \boldsymbol{\xi}\in \boldsymbol{H}(\omega). $$

An application of Theorem 3.2.1 of [14] (see also Theorem 4.4.20 of [4]) yields

11 $$  \|\boldsymbol{\xi}-\boldsymbol{{\varPi}}_{h} \boldsymbol{\xi}\| \le C h \|\boldsymbol{\xi}\|_{\omega}, $$

for all ***ξ*** ∈***H***(*ω*) ∩***V***(*ω*).

In order to provide the required estimates for the convergence of the numerical scheme, we make use of *enriching operators*. Enriching operators were first introduced in [2] (see also [1], [3], and [5]) and they play a key role in the study of obstacle problems for clamped plates (see [7] and [6]). Following Example 2.2 of [7], we recall that any enriching operator associated with conforming finite elements coincides with the canonical injection. We are going to connect Morley’s triangle to the Hsieh-Clough-Tocher macro-element (from now onwards *HCT macro-element*), that we sketch below for sake of clarity, via an ad hoc enriching operator (for a complete overview on the properties of these finite elements and the meaning of the graphical symbols used for representing the various degrees of freedom, see Figures 6.1.3 and 6.2.3 of [14]).

**Fig. 1:**
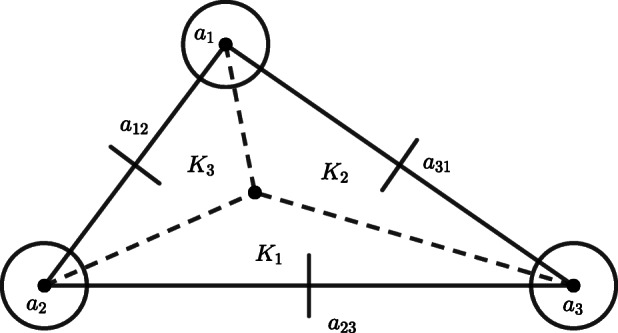
Morley’s triangle. Figure 6.2.3 of [14]

**Fig. 2:**
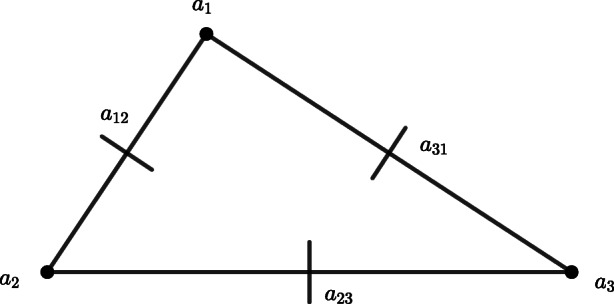
HCT macro-element. Figure 6.1.3 of [14]

The relation between the elements in Figs. 1 and 2 is due to the disposition of the vertices at which the pointwise evaluation of the shape functions occurs. Let us denote *W*_3,*h*_ the finite element space associated with the HCT macro-element and let us observe that, by the unisolvence of the HCT macro-element (cf. Theorem 6.1.2 of [14]), the elements of *W*_3,*h*_ are completely determined by their values at the vertices, the values of their first derivatives at the vertices and the values of their normal derivatives at the midpoints of the sides of the triangular element. The reason why we have to make use of a nonconforming finite element to carry out the numerical analysis of the considered obstacle problem is due to the fact that it is not natural to assume the transverse component of the solution, i.e., the one which is affected by the geometrical constraint associated with the obstacle, to be more regular than *H*^3^(*ω*) (see, for instance, [26] and [27], [9], [10], [28], and [7]).

Let us thus define the enriching operator *E*_*h*_ : *V*_3,*h*_ → *W*_3,*h*_ by (cf. formula (3.2) of [5])

12 $$  [N(E_{h} \eta)]=\frac{1}{|\mathcal{T}_{p}|}\sum\limits_{T \in \mathcal{T}_{p}} (N \eta_{T}), $$

where $p \in \mathcal {V}_{h}$ is any nodal point of the triangulation $\mathcal {T}_{h}$, *N* is any degree of freedom of the HCT macro-element associated with the nodal point *p* and $\mathcal {T}_{p}$ is the set of triangles in $\mathcal {T}_{h}$ sharing the nodal point *p*.

Next, following [3], we organize the proof of the already well-known properties of enriching operators in a series of lemmas (Lemmas 1–4).

**Fig. 3:**
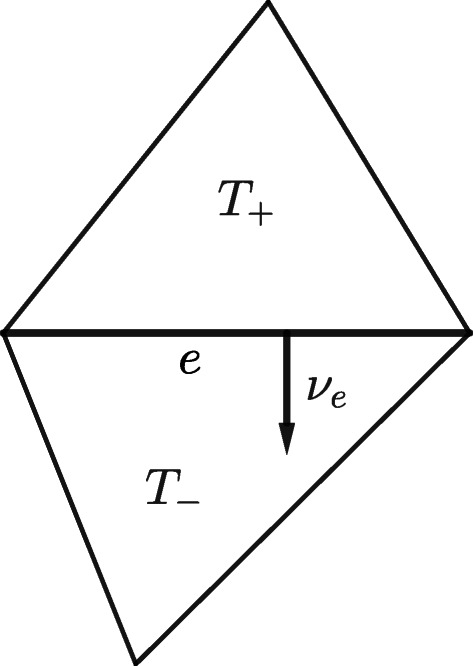
Configuration associated with the jump of the normal derivative across the edge *e*

Let us recall the definition of *jump of the normal derivative across the edge e*. Let *η* ∈ *H*^2^(*ω*) and let $e \in \mathcal {E}_{h}$ such that *e* ⊂ *ω*. The *jump of the normal derivative of η* across the edge *e* is defined as follows

13 $$  [\kern-2.5pt[ \partial_{\nu}\eta]\kern-2.5pt]:= \frac{\partial \eta_{+}}{\partial{\nu}_{e}}\bigg|_{e} - \frac{\partial \eta_{-}}{\partial{\nu}_{e}}\bigg|_{e}, $$

where *T*_+_ and *T*_−_ are elements of $\mathcal {T}_{h}$ that share the edge *e*, *η*_±_ is the restriction of *η* to *T*_±_ and *ν*_*e*_ points from *T*_+_ to *T*_−_ (see Fig. 3 below).

If *e* ⊂ *γ*, then we define the jump in this fashion:

14 $$  [\kern-2.5pt[ \partial_{\nu}\eta]\kern-2.5pt]:= -\left.\frac{\partial \eta}{\partial{\nu}_{e}}\right|_{e}. $$

The proof of the next lemma relies on standard inverse estimates (cf., e.g., [14]) and inverse trace inequalities (cf. formula (10.3.9) of [4]).

### **Lemma 1**

There exists a positive constant *C* such that

15 $$ \begin{array}{@{}rcl@{}} \|\eta-E_{h} \eta\|_{0,\omega} &\le& C h^{2} \|\eta\|_{h}\quad\text{ for all }\eta\in V_{3,h}, \end{array} $$

16 $$ \begin{array}{@{}rcl@{}} |E_{h} \eta|_{2,\omega} &\le& C \|\eta\|_{h},\qquad\text{ for all }\eta\in V_{3,h} . \end{array} $$

### *Proof*

Let us fix an arbitrary $T \in \mathcal {T}_{h}$ and let $\mathcal {N}$ denote the set of degrees of freedom of the HCT macro-element *T* (cf. Fig. 2). For any *η* ∈ *V*_3,*h*_, we have that $(\eta - E_{h} \eta )|_{K_{i}}$ is an element of $\mathbb {P}_{3}(K_{i})$, for all 1 ≤ *i* ≤ 3, where *K*_*i*_ is a sub-triangle of the HCT macro-element *T* (cf. Fig. 2). Using the same argument as in Theorem 3.1.5 of [14], we infer the existence of a positive constant *C* for which

17 $$  \|\xi\|_{0,T}^{2} \le C h^{2(1+|N|)}\sum\limits_{N \in \mathcal{N}}|N(\xi)|^{2}, $$

for all $\xi \in \mathcal {C}^{1}(T)$ such that $\xi |_{K_{i}} \in \mathbb {P}_{3}(K_{i})$, for all 1 ≤ *i* ≤ 3, where *K*_*i*_ is a sub-triangle of the HCT macro-element *T* (cf. Fig. 2) and |*N*| denotes the order of differentiation of the corresponding degree of freedom. In view of Remark 3.1.3 of [14], it results that the diameter of *T* is of order *O*(*h*) and, therefore, |*e*| = *O*(*h*) as well. By (12) and the continuity of *η* at the vertices of the triangulation, it results

$$ N(\eta)=N(E_{h} \eta)\quad\text{ if }|N|=0. $$

Therefore, letting *ξ* = *η* − *E*_*h*_*η* in (17) yields

18 $$  \|\eta-E_{h}\eta\|_{0,T}^{2} \le C h^{4} \underset{|N|=1}{\sum\limits_{N \in \mathcal{N}}} |N(\eta- E_{h} \eta)|^{2}. $$

Let us observe that if *N* is associated with the degree of freedom corresponding to the outer unit normal derivative at the midpoint of a side of the boundary *γ* then *N*(*η* − *E*_*h*_*η*) = 0.

Let *N* denote the degree of freedom corresponding to the evaluation of the outer unit normal derivative at the midpoint *m*_*e*_ of an edge *e* ⊂ *ω* and let *ν*_*e*_ denote one of the outer unit normal vectors to the edge *e*. By virtue of (12), a standard inverse estimate (Theorem 3.2.6 of [14] with $q=\infty $, *m* = *l* = 2 and *r* = 2) and an inverse trace inequality, we obtain

19 $$ \begin{array}{@{}rcl@{}} |N(\eta-E_{h} \eta)|^{2}&=&\left|\partial_{\nu_{e}}\left( \eta-E_{h}\eta\right)(m_{e})\right|^{2}\\ &=&\left[\partial_{\nu_{e}}\eta(m_{e})-\partial_{\nu_{e}}E_{h}\eta(m_{e})\right]^{2}\\ &=&\left[\frac{1}{2}\partial_{\nu_{e}}\eta_{+}(m_{e})-\frac{1}{2}\partial_{\nu_{e}}\eta_{-}(m_{e})\right]^{2}\\ &\le& \frac{|e|}{4}\|[\kern-2.5pt[\partial_{\nu_{e}}\eta]\kern-2.5pt]\|_{0,e} \le C\sum\limits_{T^{\prime} \in \mathcal{T}_{p}} |\eta|_{2,T^{\prime}}^{2}. \end{array} $$

Let *N* be the degree of freedom associated with the evaluation of any first-order derivative at any vertex $p \in \mathcal {V}_{h}$. An arithmetic-geometric mean inequality yields

20 $$  \begin{array}{llll} |N(\eta-E_{h} \eta)|^{2} &\le |\nabla(\eta-E_{h}\eta)(p)|^{2}\\ &\le C \underset{T^{\prime} \text{\tiny and }T^{\prime\prime} \text{\tiny share an edge}}{\sum\limits_{{T^{\prime}, T^{\prime\prime} \in \mathcal{T}_{p}}}}\!\!\!\!\!\!\!|\nabla \eta_{T^{\prime}}(p)-\nabla \eta_{T^{\prime\prime}}(p)|^{2}. \end{array} $$

An application of the mean value theorem (cf., e.g., Theorem 7.2-1 of [19]) like on page 915 of [2], standard inverse estimates (Theorem 3.2.6 of [14] with *r* = *m* = *l* = 2 and $q=\infty $), an inverse trace inequality, and the regularity of the triangulation gives

21 $$ \begin{array}{@{}rcl@{}} |\nabla \eta_{T^{\prime}}(p)-\nabla \eta_{T^{\prime\prime}}(p)|^{2}&\le& C|\partial_{\nu_{e}}\eta_{T^{\prime}}(p)-\partial_{\nu_{e}}\eta_{T^{\prime\prime}}(p)|^{2}\\ && + C|\partial_{\tau_{e}}\eta_{T^{\prime}}(p)-\partial_{\tau_{e}}\eta_{T^{\prime\prime}}(p)|^{2}\\ &\le& C|e|^{-1}\|[\kern-2.5pt[\partial_{\nu}\eta]\kern-2.5pt]\|_{0,e}^{2}\\ &&+C|e|^{2}(|\eta_{T^{\prime}}|_{2,\infty,T^{\prime}}^{2}+|\eta_{T^{\prime\prime}}|_{2,\infty,T^{\prime\prime}}^{2})\\ &\le& C\sum\limits_{T^{\prime} \in \mathcal{T}_{e}}|\eta|_{2,T^{\prime}}^{2}, \end{array} $$

where $\mathcal {T}_{e}$ is the set of triangles sharing the edge *e*. Combining (18)–(21), we obtain

22 $$  \|\eta-E_{h}\eta\|_{0,T}^{2} \le C h^{4} \sum\limits_{T^{\prime} \in \mathcal{T}_{T}} |\eta|_{2,T^{\prime}}^{2}. $$

Estimate (15) follows by summing up (22) over all the triangles of $\mathcal {T}_{h}$. Estimate (16) follows by standard inverse estimates (Theorem 3.2.6 of [14] with *m* = 2, *l* = 0, *p* = *r* = 2) and (22): Indeed,

$$ \begin{array}{@{}rcl@{}} |E_{h} \eta|_{2,\omega}^{2}&\le& C \sum\limits_{T \in \mathcal{T}_{h}}|\eta-E_{h}\eta|_{2,T}^{2}+\sum\limits_{T \in \mathcal{T}_{h}}|\eta|_{2,T}^{2}\\ &\le& \sum\limits_{T \in \mathcal{T}_{h}}\left[h^{-4}\|\eta-E_{h} \eta\|_{0,T}^{2}+|\eta|_{2,T}^{2}\right] \le C \|\eta\|_{h}^{2}. \end{array} $$

This completes the proof. □

By virtue of an interpolation estimate (see, for instance, Theorem 3.1.5 of [14] with *m* = *q* = *p* = *k* = 2), which holds by the fact that Morley’s triangle is *almost affine* (cf., e.g., [33]), and the standard trace theorem for Sobolev spaces defined over domains, we deduce that, for all $T\in \mathcal {T}_{h}$,

23 $$  \|\nabla(\eta-{\varPi}_{3,h}\eta)\|_{0,\partial T} \le C h |\eta|_{3,T}, $$

where *η* ∈ *H*^3^(*T*) ∩ *V*_3_(*ω*). By (12), we deduce that *E*_*h*_*η* = *η* at the internal nodes of the triangulation (see also formula (6.11) of [3]). As a result,

$$ {\varPi}_{3,h} E_{h} \eta=\eta\quad\text{ for all }\eta\in V_{3,h}. $$

The next preliminary result is inspired by Lemma 2 of [5], which is itself based on the unisolvence of the HCT macro-element (see, for instance, Theorem 6.1.2 of [14]) and Bramble-Hilbert lemma (cf., e.g., Theorem 4.1.3 of [14]). For convenience, we provide a complete proof.

### **Lemma 2**

**Fig. 4:**
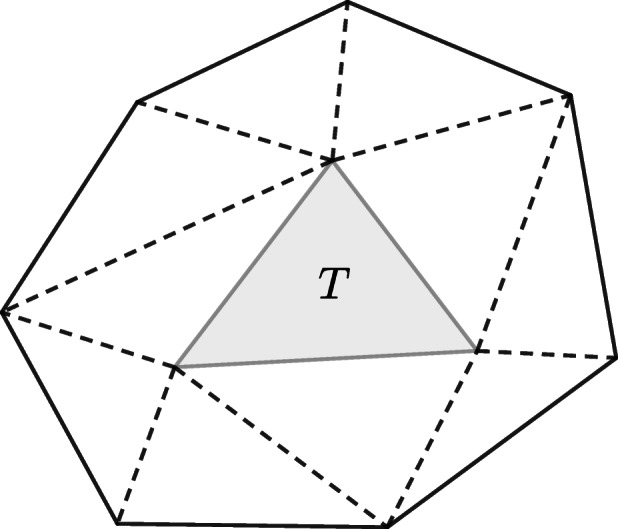
The polygon *S*_*T*_ made of all the triangles of $\mathcal {T}_{T}$. Figure 9 of [1]

There exists a positive constant *C* solely depending on the regularity of the triangulation $\mathcal {T}_{h}$ such that

24 $$  \sum\limits_{m=0}^{^{2}}h^{2m} |\eta-E_{h} {\varPi}_{3,h}\eta|_{m,T}^{2} \le C h^{6}|\eta|_{3,S_{T}}^{2}, $$

for all $T \in \mathcal {T}_{h}$ and all *η* ∈ *H*^3^(*S*_*T*_) ∩ *H*^2^(*ω*), where *S*_*T*_ is the polygon formed by all the triangles of $\mathcal {T}_{h}$ sharing a vertex with *T* (cf. Fig. 4 below).

### *Proof*

Let $T \in \mathcal {T}_{h}$ be an arbitrary element. Then, we observe that (see Lemma 2 of [5]) the expression (*E*_*h*_*π*_3,*h*_*η*)|_*T*_ is completely determined by $\eta |_{S_{T}}$ and that the mapping

$$ \eta|_{S_{T}} \mapsto (\eta-E_{h}{\varPi}_{3,h}\eta)|_{T}, $$

is bounded from *H*^3^(*S*_*T*_) into *H*^2^(*T*).

Moreover, (12) and the unisolvence of the HCT macro-element give

25 $$  q-E_{h} {\varPi}_{3,h}q=0\quad\text{ for all }q \in \mathbb{P}_{2}(T). $$

Thanks to (25), we can apply the Bramble-Hilbert lemma and infer the validity of (24). The proof is thus complete. □

We now modify the definition of the enriching operator *E*_*h*_ in order to *incorporate the boundary conditions*. As a result, we obtain the corresponding enriching operator $\tilde {E}_{h}:\tilde {V}_{3,h}\to \tilde {W}_{3,h}$, where $\tilde {V}_{3,h}$ denotes the subspace of *V*_3,*h*_ whose degrees of freedom vanish along *γ* and $\tilde {W}_{3,h}:=W_{3,h}\cap {H^{2}_{0}}(\omega )$, i.e., the subspace of *W*_3,*h*_ whose degrees of freedom vanish along *γ* (cf. Example 6.1 of [3] and [6]). Observe that *E*_*h*_*η* ∈ *H*^2^(*ω*) by the properties of the HCT macro-element. We now define $\tilde {E}_{h}$ as follows, in such a way that $\tilde {E}_{h} \eta \in {H^{2}_{0}}(\omega )$ for all $\eta \in \tilde {V}_{3,h}$:

(i) The degrees of freedom of *E*_*h*_*η* and $\tilde {E}_{h}\eta $ coincide in *ω*,
(ii) The degrees of freedom of $\tilde {E}_{h} \eta $ vanish on *γ*.

In the next lemma, we prove the first properties of the modified enriching operator $\tilde {E}_{h}$. The proof resorts to standard inverse estimates (cf., e.g., [14]), an inverse trace inequality (cf. formula (10.3.9) of [4]) and Lemma 1.

### **Lemma 3**

For each $\eta \in \tilde {V}_{3,h}$, there exists a positive constant *C* such that

26 $$ \begin{array}{@{}rcl@{}} \|\eta-\tilde{E}_{h}\eta\|_{0,\omega}&\le& C h^{2} \|\eta\|_{h}, \end{array} $$

27 $$ \begin{array}{@{}rcl@{}} \|\tilde{E}_{h} \eta\|_{2,\omega} &\le& C \|\eta\|_{h}. \end{array} $$

### *Proof*

Let us fix an arbitrary element $\eta \in \tilde {V}_{3,h}$ and let *N* be any degree of freedom associated with the HCT macro-element. Let us observe that if *N* is not related to any nodal point of *γ*, then, by property (*i*) in the definition of $\tilde {E}_{h}$, we obtain

$$ N(E_{h} \eta-\tilde{E}_{h}\eta)=0. $$

On the other hand, for all $T \in \mathcal {T}_{h}$, if |*N*| = 0 and *N* is related to a nodal point of *γ*, then, by property (*ii*) in the definition of $\tilde {E}_{h}$ and the fact that $\eta \in \tilde {V}_{3,h}$, it follows that

$$ N(E_{h} \eta-\tilde{E}_{h}\eta)=N(E_{h}\eta)=0. $$

Let *m*_*e*_ be the midpoint of an edge *e* ⊂ *γ*. By (14) and standard inverse estimates (Theorem 3.2.6 of [14] with *m* = *l* = 0, $q=\infty $ and *r* = 2), we obtain

28 $$  |\partial_{\nu_{e}}(E_{h}\eta-\tilde{E}_{h}\eta)(m_{e})|^{2}=|\partial_{\nu_{e}}\eta(m_{e})|^{2}\le \frac{C}{|e|}\|[\kern-2.5pt[\partial_{\nu}\eta]\kern-2.5pt]\|_{0,e}^{2}\le C \sum\limits_{T^{\prime} \in \mathcal{T}_{e}}|\eta|_{2,T^{\prime}}^{2}, $$

where the latter inequality holds true by virtue of an inverse trace inequality and the regularity of the triangulation $\mathcal {T}_{h}$.

Let *p* be a vertex on *γ*. Then, *p* is the endpoint of an edge *e*^∗^⊂ *γ*. Let *T*^∗^ be an element of $\mathcal {T}_{h}$ such that *e*^∗^⊂ *T*^∗^. By (20), (21), an arithmetic-geometric mean inequality, standard inverse estimates (Theorem 3.2.6 of [14] with *m* = *l* = *r* = 2 and $q=\infty $), and the regularity of the triangulation, we get

29 $$  \begin{array}{llll} |\nabla(E_{h}\eta-\tilde{E}_{h}\eta)(p)|^{2}&=|\nabla(E_{h}\eta)(p)|^{2}\\ &\le C (|\nabla(E_{h}\eta-\eta_{T^{\ast}})(p)|^{2}+|\nabla \eta_{T^{\ast}}(p)|^{2})\\ &\le C \sum\limits_{T^{\prime} \in \mathcal{T}_{T}}|\eta|_{2,T^{\prime}}^{2}, \end{array} $$

where $\mathcal {E}_{p}$ is the set of edges of $\mathcal {E}_{h}$ sharing *p* as a common vertex.

In conclusion, for all $T \in \mathcal {T}_{h}$, a combination of (22), (28), and (29) yields

30 $$  \|\eta-\tilde{E}_{h}\eta\|_{0,T}^{2}\le C h^{4} {\sum}_{T^{\prime} \in \mathcal{T}_{T}}|\eta|_{2,T^{\prime}}^{2}, $$

which implies the inequality (26) after a summation over all the elements of $\mathcal {T}_{h}$.

Using the Poincaré-Friedrichs inequality, an arithmetic-geometric mean inequality, (26) and standard inverse estimates (Theorem 3.2.6 of [14] with *m* = 2, *l* = 0, *q* = *r* = 2), we infer the validity of (27). Indeed,

$$ \begin{array}{@{}rcl@{}} \|\tilde{E}_{h} \eta\|_{2,\omega}^{2} &\le& C |\tilde{E}_{h} \eta|_{2,\omega}^{2} \le \sum\limits_{T \in \mathcal{T}_{h}} \left[ |\eta-\tilde{E}_{h}\eta|_{2,T}^{2}+ |\eta|_{2,T}^{2}\right]\\ &\le&\sum\limits_{T \in \mathcal{T}_{h}}\left[h^{-4}\|\eta-\tilde{E}_{h}\eta\|_{0,T}^{2}+|\eta|_{2,T}^{2}\right] \le C \|\eta\|_{h}^{2}, \end{array} $$

which completes the proof. □

By property (*i*) in the definition of $\tilde {E}_{h}$, we can easily observe that the following holds (cf. [5]):

$$ {\varPi}_{3,h} \tilde{E}_{h} \eta=\eta\quad\text{ for all }\eta\in \tilde{V}_{3,h}. $$

The next step consists in incorporating the boundary conditions into the above estimates.

### **Lemma 4**

There exists a positive constant *C* solely depending on the regularity of the triangulation $\mathcal {T}_{h}$ such that

31 $$  \sum\limits_{m=0}^{2} h^{2m}|\eta-\tilde{E}_{h}{\varPi}_{3,h}\eta|_{m,T}^{2} \le C h^{6} |\eta|_{3,S_{T}}^{2}, $$

for all $T\in \mathcal {T}_{h}$ and all *η* ∈ *H*^3^(*S*_*T*_) ∩ *V*_3_(*ω*).

### *Proof*

Let us fix an arbitrary element $\eta \in \tilde {V}_{3,h}$ and let *N* be any degree of freedom associated with an internal vertex of the triangulation made of HCT macro-element. Like in Lemma 3, we have

$$ N(E_{h} \eta-\tilde{E}_{h}\eta)=0. $$

Let $e\in \mathcal {E}_{h}$ be an edge contained in *γ* and let *m*_*e*_ be the midpoint of *e*, at which the normal derivative of *η* is evaluated. By virtue of (14), (23), (28), and a standard inverse estimate (Theorem 3.1.5 of [14] with *m* = *q* = *k* = *p* = 2), we obtain

32 $$ \begin{array}{@{}rcl@{}} |\partial_{\nu_{e}}(E_{h}{\varPi}_{3,h}\eta-\tilde{E}_{h}{\varPi}_{3,h}\eta)(m_{e})|^{2}&=& |\partial_{\nu_{e}}(E_{h}{\varPi}_{3,h}\eta)(m_{e})|^{2}\\ &=&|\partial_{\nu_{e}}({\varPi}_{3,h}\eta)(m_{e})|^{2}\\ &\le& C|e|^{-1}\|[\kern-2.5pt[\partial_{\nu}{\varPi}_{3,h}\eta]\kern-2.5pt]\|_{0,e}^{2} \\ &\le& C h^{2} \sum\limits_{T^{\prime} \in \mathcal{T}_{e}} |\eta|_{3,T^{\prime}}^{2}, \end{array} $$

for all *η* ∈ *H*^3^(*S*_*T*_) ∩ *V*_3_(*ω*).

Similarly, for any vertex *p* ∈ *γ*, we have, by (29), standard interpolation estimates (Theorem 3.1.5 of [14]) and an inverse trace inequality (formula (10.3.9) of [4])

33 $$ \begin{array}{@{}rcl@{}} |\nabla(E_{h} {\varPi}_{3,h} \eta-\tilde{E}_{h} {\varPi}_{3,h} \eta)(p)|^{2}&\le& \sum\limits_{T \in \mathcal{T}_{p}} |\partial_{\tau_{e}}(E_{h} {\varPi}_{3,h} \eta_{T})(p)|^{2}\\ &&+\sum\limits_{T \in \mathcal{T}_{p}} |\partial_{\nu_{e}}(E_{h} {\varPi}_{3,h} \eta_{T})(p)|^{2}\\ &\le& C \sum\limits_{T \in \mathcal{T}_{p}} |\partial_{\tau_{e}}({\varPi}_{3,h} \eta_{T} -\eta_{T})(p)|^{2}\\ &&+C \sum\limits_{e \in \mathcal{E}_{p}}|e|^{-1} \|[\kern-2.5pt[\partial_{\nu}{\varPi}_{3,h}\eta]\kern-2.5pt]\|_{0,e}^{2}\\ &\le& C \sum\limits_{T \in \mathcal{T}_{p}} |{\varPi}_{3,h} \eta_{T} -\eta_{T}|_{2,T}^{2}\\ &&+C \sum\limits_{e \in \mathcal{E}_{p}}|e|^{-1} \|[\kern-2.5pt[\partial_{\nu}({\varPi}_{3,h}\eta-\eta)]\kern-2.5pt]\|_{0,e}^{2}\\ &\le& C h^{2} \sum\limits_{T \in \mathcal{T}_{p}} |\eta|_{3,T}^{2}. \end{array} $$

Summing over all the triangles of $\mathcal {T}_{h}$, we obtain the following estimate

34 $$  \begin{array}{llll} &\sum\limits_{m=0}^{2} h^{2m}|\eta-E_{h}{\varPi}_{3,h}\eta|_{m,T}^{2} \le Ch^{4}\underset{|N|=1}{\sum\limits_{N \in \mathcal{N}}}|N(\eta-E_{h} {\varPi}_{3,h}\eta)|^{2}\\ &\quad + \sum\limits_{m=1}^{2} h^{2m} |\eta-E_{h}{\varPi}_{3,h}\eta|_{m,T}^{2} \le C h^{6} |\eta|_{3,S_{T}}^{2}, \end{array} $$

where the first inequality holds by (18) and the latter inequality holds by (32) and (33). This completes the proof. □

As a consequence of Lemmas 1–4 and standard inverse estimates (Theorem 3.2.6 of [14] with *m* = 1, *l* = 0, *q* = *r* = 2), we obtain the following estimates for the enriching operator $\tilde {E}_{h}$ (cf. Corollary 1 of [5]):

35 $$ \begin{array}{@{}rcl@{}} &&\|\eta-\tilde{E}_{h}\eta\|_{0,\omega}+h\left( \sum\limits_{T \in \mathcal{T}_{h}}|\eta-\tilde{E}_{h}\eta|_{1,T}^{2}\right)^{1/2}+h^{2}|\tilde{E}_{h} \eta|_{2,\omega}\\ &&\quad\le C h^{2}\|\eta\|_{h}\quad\text{ for all }\eta\in \tilde{V}_{3,h}, \end{array} $$

36 $$ \begin{array}{@{}rcl@{}} &&\sum\limits_{m=0}^{2} h^{m}|\eta-\tilde{E}_{h}{\varPi}_{3,h}\eta|_{m,\omega} \le C h^{3}|\eta|_{3,\omega}\quad\text{ for all }\eta \in H^{3}(\omega)\cap V_{3}(\omega). \end{array} $$

Define the space

$$ \begin{array}{@{}rcl@{}} \tilde{\boldsymbol{W}}_{h}&:= V_{1,h}\times V_{2,h}\times \tilde{W}_{3,h}, \end{array} $$

and let us define the enriching operator $\tilde {\boldsymbol {E}}_{h}:\tilde {\boldsymbol {V}}_{h} \to \tilde {\boldsymbol {W}}_{h}$ as follows:

37 $$  \tilde{\boldsymbol{E}}_{h} \boldsymbol{\xi}:=(\xi_{1},\xi_{2}, \tilde{E}_{h} \xi_{3})\quad\text{ for all }\boldsymbol{\xi}\in\tilde{\boldsymbol{V}}_{h}. $$

A direct application of (35) and (36) to (37) yields

38 $$ \begin{array}{@{}rcl@{}} \begin{array}{ll} &\|\boldsymbol{\eta}-\tilde{\boldsymbol{E}}_{h}\boldsymbol{\eta}\|_{0,\omega}+h\left( \sum\limits_{T \in \mathcal{T}_{h}}|\boldsymbol{\eta}-\tilde{\boldsymbol{E}}_{h}\boldsymbol{\eta}|_{1,T}^{2}\right)^{1/2}+h^{2}|\tilde{\boldsymbol{E}}_{h} \boldsymbol{\eta}|_{2,\omega}\\ &\quad\le C h^{2}\|\boldsymbol{\eta}\|\quad\text{ for all }\boldsymbol{\eta}\in \tilde{\boldsymbol{V}}_{h}. \end{array} \end{array} $$

Next, we prove a crucial estimate for *b*_*h*_(⋅,⋅) in the case where the transverse component of the displacement is approximated via Morley’s triangles. The assumption that the solution ***ζ***^*ε*^ to Problem $\mathcal {P}^{\varepsilon }(\omega )$ is “more regular” is of paramount importance.

By virtue of the results proved in [9], [10], [27], and [28] and in order to make our analysis more general, we will derive the sought error estimate under the constraint that the transverse component of the solution of Problem $\mathcal {P}^{\varepsilon }(\omega )$ cannot be more regular than *H*^3^(*ω*).

In order to derive error estimate, we will have to assume that the solution of Problem $\mathcal {P}^{\varepsilon }(\omega )$ is more regular (cf., e.g., [14]); in particular, we will assume that

$$ \boldsymbol{\zeta}^{\varepsilon} \in \boldsymbol{H}(\omega)\cap \boldsymbol{V}(\omega). $$

The augmented regularity result for the tangential components is studied, for instance, in Section 8.7 of Chapter 2 of [38], while the augmented regularity result for the transverse component is given for solutions of some fourth-order variational inequalities on pages 323–327 of [30], and is also recalled in [45].

To prove the next result we follow Appendix B of [7] and Lemma 4.2 of [6]. As a consequence of the trace properties (cf., e.g., Theorem 6.6-5 of [19]), we can take into account the average along any edge $e \in \mathcal {E}_{h}$ of a function *f* ∈ *H*^1^(*ω*) and denote it by $\overline {f}$, viz.,

$$ \overline{f}:=\frac{1}{|e|}{\int}_{e} f \mathrm{d} s \in \mathbb{R}. $$

### **Lemma 5**

There exists a positive constant *C* such that the following estimate holds

39 $$  |b_{h}(\boldsymbol{\zeta}^{\varepsilon}, \boldsymbol{\eta}-\tilde{\boldsymbol{E}}_{h}\boldsymbol{\eta})|\le Ch \|\boldsymbol{\zeta}^{\varepsilon}\|_{\omega}\|\boldsymbol{\eta}\|\quad\text{ for all }\boldsymbol{\eta}\in \tilde{\boldsymbol{V}}_{h}, $$

where ***ζ***^*ε*^ ∈***H***(*ω*) ∩***V***(*ω*) is the solution to Problem $\mathcal {P}^{\varepsilon }(\omega )$.

### *Proof*

Observe that if $\eta _{3} \in \tilde {V}_{3,h}$ then ∇*η*_3_ is continuous at the midpoints of the edges $e \in \mathcal {E}_{h}$ and vanishes at the midpoints of the edges along *γ*. Indeed, after fixing an edge $e \in \mathcal E_{h}$, consider the restrictions $\eta _{3}|_{{T}_{+}}$ and $\eta _{3}|_{{T}_{-}}$ to the edge *e*, where *T*_±_ are, again, the elements of $\mathcal {T}_{h}$ that share the edge *e*. Then, $(\eta _{3}|_{{T}_{+}}-\eta _{3}|_{{T}_{-}}) \in \mathbb {P}_{2}(\mathbb {R}^{2})$ and $(\eta _{3}|_{{T}_{+}}-\eta _{3}|_{{T}_{-}})$ vanishes at the endpoints of the edge *e*. As a result, by the mean value theorem and the properties of quadratic polynomials, we deduce that the *tangential derivative along the edge e* $\partial _{\tau _{e}}(\eta _{3}|_{{T}_{+}}-\eta _{3}|_{{T}_{-}})$ vanishes at the midpoint of *e*. By virtue of the decomposition of the gradient in terms of tangential and normal derivatives we get the continuity of the gradient at the midpoint of any edge $e \in \mathcal {E}_{h}$. The other property follows from the boundary conditions.

Combining the definition and the properties of $\tilde {\boldsymbol {E}}_{h}$, Green’s formula, the midpoint rule, the Cauchy-Schwarz inequality, inverse trace inequalities (formulas (10.3.8) and (10.3.9) of [4]), the Poincaré-Friedrichs inequality (Theorems 6.5-2 and 6.8-1 of [19]), and (38), we obtain

$$ \begin{array}{@{}rcl@{}} b_{h}(\boldsymbol{\zeta}^{\varepsilon}, \boldsymbol{\eta}-\tilde{\boldsymbol{E}}_{h}\boldsymbol{\eta})&=&-\sum\limits_{T \in \mathcal{T}_{h}}{\int}_{T} m_{\alpha\beta}^{\varepsilon}(\zeta_{3}^{\varepsilon}) \partial_{\alpha\beta}(\eta_{3}-\tilde{E}_{h}\eta_{3}) \mathrm{d} y\\ &&+\sum\limits_{T \in \mathcal{T}_{h}}{\int}_{T} (\partial_{\alpha} \theta^{\varepsilon}) n_{\alpha\beta}^{\theta,\varepsilon}(\boldsymbol{\zeta}^{\varepsilon}) \partial_{\beta}(\eta_{3}-\tilde{E}_{h} \eta_{3}) \mathrm{d} y\\ &=&\sum\limits_{T \in \mathcal{T}_{h}}{\int}_{T} \partial_{\alpha}(m_{\alpha\beta}^{\varepsilon}(\zeta_{3}^{\varepsilon})) \partial_{\beta}(\eta_{3}-\tilde{E}_{h}\eta_{3}) \mathrm{d} y\\ &&-\sum\limits_{T \in \mathcal{T}_{h}} {\int}_{\partial T}m_{\alpha\beta}^{\varepsilon}(\zeta_{3}^{\varepsilon}) \partial_{\beta}(\eta_{3}-\tilde{E}_{h}\eta_{3}) \nu_{\alpha} \mathrm{d} s\\ &&+\sum\limits_{T \in \mathcal{T}_{h}} {\int}_{T} (\partial_{\alpha} \theta^{\varepsilon}) n_{\alpha\beta}^{\theta,\varepsilon}(\boldsymbol{\zeta}^{\varepsilon}) \partial_{\beta}(\eta_{3}-\tilde{E}_{h} \eta_{3}) \mathrm{d} y\\ &=&\sum\limits_{T \in \mathcal{T}_{h}} {\int}_{T} \left[\partial_{\alpha}(m_{\alpha\beta}^{\varepsilon}(\zeta_{3}^{\varepsilon}))+(\partial_{\alpha} \theta^{\varepsilon}) n_{\alpha\beta}^{\theta,\varepsilon}(\boldsymbol{\zeta}^{\varepsilon}) \right]\partial_{\beta}(\eta_{3}-\tilde{E}_{h}\eta_{3}) \mathrm{d} y\\ &&- \sum\limits_{e \in \mathcal{E}_{h}}{\int}_{e}\left( m_{\alpha\beta}^{\varepsilon}(\zeta_{3}^{\varepsilon})-\overline{m_{\alpha\beta}^{\varepsilon}(\zeta_{3}^{\varepsilon})}\right) [\kern-2.5pt[\partial_{\beta}(\eta_{3}-\tilde{E}_{h}\eta_{3}) \nu_{\alpha}]\kern-2.5pt] \mathrm{d} s\\ &\le& \sum\limits_{T \in \mathcal{T}_{h}}\left( \|\partial_{\alpha} m_{\alpha\beta}^{\varepsilon}(\zeta_{3}^{\varepsilon})+(\partial_{\alpha} \theta^{\varepsilon}) n_{\alpha\beta}^{\theta,\varepsilon}(\boldsymbol{\zeta}^{\varepsilon})\|_{0,T} \|\partial_{\beta}(\eta_{3}-\tilde{E}_{h}\eta_{3})\|_{0,T}\right)\\ &&+\left( \sum\limits_{e \in \mathcal{E}_{h}} |e|^{-1} \|m_{\alpha\beta}^{\varepsilon}(\zeta_{3}^{\varepsilon})-\overline{m_{\alpha\beta}^{\varepsilon}(\zeta_{3}^{\varepsilon})}\|_{0,e}\right)^{1/2}\\ &&\times \left( \sum\limits_{e \in \mathcal{E}_{h}} |e| \|[\kern-2.5pt[\partial_{\beta}(\eta_{3}-\tilde{E}_{h}\eta_{3}) \nu_{\alpha}]\kern-2.5pt]\|_{0,e}\right)^{1/2}\\ &\le& C \|\boldsymbol{\zeta}^{\varepsilon}\|_{\omega} \left( \sum\limits_{T \in \mathcal{T}_{h}}|\eta_{3}-\tilde{E}_{h}\eta_{3}|_{1,T}^{2}\right)^{1/2} \le C h \|\boldsymbol{\zeta}^{\varepsilon}\|_{\omega} \|\boldsymbol{\eta}\|, \end{array} $$

where, in analogy with (13) and (14), we have

$$ [\kern-2.5pt[\partial_{\beta}(\eta_{3}-\tilde{E}_{h}\eta_{3}) \nu_{\alpha}]\kern-2.5pt]:= (\partial_{\beta}(\eta_{3}-\tilde{E}_{h}\eta_{3}) \nu_{\alpha,+})+(\partial_{\beta}(\eta_{3}-\tilde{E}_{h}\eta_{3}) \nu_{\alpha,-}), $$

which completes the proof. □

Let us observe that, by virtue of the definition of $\tilde {\boldsymbol {E}}_{h}$ (cf. (37)), the variational equations (6) do not give any contribution in the previous proof .

Having extended the properties of the enriching operator to our problem, we now prove, following [7], a series of preparatory lemmas. Let us recall that, for all *h* > 0, the symbol $\mathcal {V}_{h}$ designates the set of all of the nodal points of the triangulation $\mathcal {T}_{h}$.

Let us define the functional *J*_*h*_ and the set $K_{3,h}^{\varepsilon }$ as follows:

40 $$ \begin{array}{@{}rcl@{}} J_{h}(\boldsymbol{\eta}_{h})&:=&\frac{1}{2}b_{h}(\boldsymbol{\eta}_{h},\boldsymbol{\eta}_{h})-\ell(\boldsymbol{\eta}_{h})\quad\text{ for all }\boldsymbol{\eta}_{h} \in \boldsymbol{V}_{h}, \end{array} $$

41 $$ \begin{array}{@{}rcl@{}} K_{3,h}^{\varepsilon}&:=&\{\eta_{3,h} \in \tilde{V}_{3,h};\theta^{\varepsilon}(p)+\eta_{3,h}(p)\ge 0 \text{ for all }p\in\mathcal{V}_{h}\}, \end{array} $$

and let us then state the *approximate problem* $\mathcal {P}_{h}^{\varepsilon }$ corresponding to Problem $\mathcal {P}^{\varepsilon }(\omega )$.

### **Problem 3**

$\mathcal {P}_{h}^{\varepsilon }$ Find $\boldsymbol {\zeta }_{h}^{\varepsilon } \in \tilde {\boldsymbol {V}}_{h}$ such that the transverse component $\zeta _{3,h}^{\varepsilon }$ belongs to $K_{3,h}^{\varepsilon }$ and such that

42 $$  J_{h}(\boldsymbol{\zeta}_{h}^{\varepsilon})=\underset{\eta_{3,h} \in K_{3,h}^{\varepsilon}}{\inf_{\boldsymbol{\eta}_{h} \in \tilde{\boldsymbol{V}}_{h}}}J_{h}(\boldsymbol{\eta}_{h}). $$

$\blacksquare $

Since the bilinear form *b*_*h*_(⋅,⋅) is symmetric and continuous over the space $\tilde {\boldsymbol {V}}_{h}$ and it is $\tilde {\boldsymbol {V}}_{h}$-elliptic, we infer that Problem $\mathcal {P}_{h}^{\varepsilon }$ has a unique solution $\boldsymbol {\zeta }_{h}^{\varepsilon }$, which satisfies the variational inequalities

43 $$  b_{h}(\boldsymbol{\zeta}_{h}^{\varepsilon}, \boldsymbol{\eta}_{h}-\boldsymbol{\zeta}_{h}) \ge \ell(\boldsymbol{\eta}_{h}-\boldsymbol{\zeta}_{h}^{\varepsilon}), $$

for all $\boldsymbol {\eta }_{h}\in \tilde {\boldsymbol {V}}_{h}$ such that $\eta _{3,h}\in K_{3,h}^{\varepsilon }$.

### **Lemma 6**

Let ***ζ***^*ε*^ and $\boldsymbol {\zeta }_{h}^{\varepsilon }$ respectively denote the solutions to Problem $\mathcal {P}^{\varepsilon }(\omega )$ and Problem $\mathcal {P}_{h}^{\varepsilon }$. There exist two constants *C*_1_ > 0 and *C*_2_ > 0 such that

44 $$  \|\boldsymbol{\zeta}^{\varepsilon}-\boldsymbol{\zeta}_{h}^{\varepsilon}\|^{2} \le C_{1} \|\boldsymbol{\zeta}^{\varepsilon}-\boldsymbol{{\varPi}}_{h}\boldsymbol{\zeta}^{\varepsilon}\|^{2}+C_{2}\left[b_{h}(\boldsymbol{\zeta}^{\varepsilon},\boldsymbol{{\varPi}}_{h}\boldsymbol{\zeta}^{\varepsilon}-\boldsymbol{\zeta}_{h}^{\varepsilon})-\ell(\boldsymbol{{\varPi}}_{h}\boldsymbol{\zeta}^{\varepsilon}-\boldsymbol{\zeta}_{h}^{\varepsilon})\right]. $$

### *Proof*

Observe that $\boldsymbol {{\varPi }}_{h} \boldsymbol {\zeta }^{\varepsilon }$ belongs to the space $\tilde {\boldsymbol {V}}_{h}$. By the continuity and the $\tilde {\boldsymbol {V}}_{h}$-ellipticity of *b*_*h*_(⋅,⋅) and Young’s inequality (see [47]), we get

$$ \begin{array}{@{}rcl@{}} \alpha\|\boldsymbol{{\varPi}}_{h}\boldsymbol{\zeta}^{\varepsilon}-\boldsymbol{\zeta}_{h}^{\varepsilon}\|^{2}&\le& b_{h}(\boldsymbol{{\varPi}}_{h}\boldsymbol{\zeta}^{\varepsilon}-\boldsymbol{\zeta}_{h}^{\varepsilon},\boldsymbol{{\varPi}}_{h}\boldsymbol{\zeta}^{\varepsilon}-\boldsymbol{\zeta}_{h}^{\varepsilon})\\ &\le& M \|\boldsymbol{{\varPi}}_{h}\boldsymbol{\zeta}^{\varepsilon}-\boldsymbol{\zeta}^{\varepsilon}\|\|\boldsymbol{{\varPi}}_{h}\boldsymbol{\zeta}^{\varepsilon}-\boldsymbol{\zeta}_{h}^{\varepsilon}\| + b_{h}(\boldsymbol{\zeta}^{\varepsilon}, \boldsymbol{{\varPi}}_{h}\boldsymbol{\zeta}^{\varepsilon}-\boldsymbol{\zeta}_{h}^{\varepsilon})-\ell(\boldsymbol{{\varPi}}_{h}\boldsymbol{\zeta}^{\varepsilon}-\boldsymbol{\zeta}_{h}^{\varepsilon})\\ &\le& \frac{M}{2}\left[\frac{M}{\alpha}\|\boldsymbol{{\varPi}}_{h}\boldsymbol{\zeta}^{\varepsilon}-\boldsymbol{\zeta}^{\varepsilon}\|^{2}+\frac{\alpha}{M}\|\boldsymbol{{\varPi}}_{h}\boldsymbol{\zeta}^{\varepsilon}-\boldsymbol{\zeta}_{h}^{\varepsilon}\|^{2}\right]\\ &&+ b_{h}(\boldsymbol{\zeta}^{\varepsilon}, \boldsymbol{{\varPi}}_{h}\boldsymbol{\zeta}^{\varepsilon}-\boldsymbol{\zeta}_{h}^{\varepsilon})-\ell(\boldsymbol{{\varPi}}_{h}\boldsymbol{\zeta}^{\varepsilon}-\boldsymbol{\zeta}_{h}^{\varepsilon}). \end{array} $$

Letting *C*_1_ := *M*^2^/*α*^2^ and *C*_2_ := *α*^− 1^, we obtain inequality (44). □

## An intermediary problem

In what follows, we shall estimate the term $[b_{h}(\boldsymbol {\zeta }^{\varepsilon }, \boldsymbol {{\varPi }}_{h}\boldsymbol {\zeta }^{\varepsilon }-\boldsymbol {\zeta }_{h}^{\varepsilon })-\ell (\boldsymbol {{\varPi }}_{h}\boldsymbol {\zeta }^{\varepsilon }-\boldsymbol {\zeta }_{h}^{\varepsilon })]$ in order to apply the interpolation estimate (11). To this aim, we introduce an *intermediary problem*, since it is not easy to directly connect $K_{3}^{\varepsilon }(\omega )$ to $K_{3,h}^{\varepsilon }$. Define the set

45 $$  \tilde{K}_{3,h}^{\varepsilon}(\omega):=\{\eta_{3} \in {H^{2}_{0}}(\omega);\theta^{\varepsilon}(p)+\eta_{3}(p)\ge 0 \text{ for all }p\in\mathcal{V}_{h}\} $$

and define

$$ \boldsymbol{V}_{h}(\omega):={H^{1}_{0}}(\omega)\times {H^{1}_{0}}(\omega)\times \tilde{K}_{3,h}^{\varepsilon}(\omega) $$

as to define the functional $J:\boldsymbol {V}_{h}(\omega ) \to \mathbb {R}$ by

46 $$  J(\boldsymbol{\eta}):=\frac{1}{2}b(\boldsymbol{\eta},\boldsymbol{\eta})-\ell(\boldsymbol{\eta}). $$

Let us state the intermediary problem $\mathcal {P}_{h}^{\varepsilon }(\omega )$ establishing the connection between Problem $\mathcal {P}^{\varepsilon }(\omega )$ and Problem $\mathcal {P}_{h}^{\varepsilon }$.

### **Problem 4**

$\mathcal {P}_{h}^{\varepsilon }(\omega )$ Find $\tilde {\boldsymbol {\zeta }}_{h}^{\varepsilon } \in \boldsymbol {V}_{h}(\omega )$ such that the transverse component $\tilde {\zeta }_{3,h}^{\varepsilon }$ belongs to $\tilde {K}_{3,h}^{\varepsilon }(\omega )$ and such that

47 $$  J(\tilde{\boldsymbol{\zeta}}_{h}^{\varepsilon})=\underset{\eta_{3} \in \tilde{K}_{3,h}^{\varepsilon}(\omega)}{\inf_{{\boldsymbol{\eta} \in \boldsymbol{V}_{h}(\omega)}}}J(\boldsymbol{\eta}). $$

$\blacksquare $

Using properties (*i*) and (*ii*) of the enriching operator $\tilde {E}_{h}$, we immediately obtain that

$$ \tilde{E}_{h} \eta_{3,h} \in \tilde{K}_{3,h}^{\varepsilon}(\omega)\quad\text{ for all }\eta_{3,h}\in K_{3,h}^{\varepsilon}. $$

Using the symmetry and the continuity and the ***V***(*ω*)-ellipticity of the bilinear form *b*(⋅,⋅), we infer that Problem $\mathcal {P}_{h}^{\varepsilon }(\omega )$ admits one and only one solution $\tilde {\boldsymbol {\zeta }}_{h}^{\varepsilon }$ satisfying the following variational inequalities:

48 $$  b(\tilde{\boldsymbol{\zeta}}_{h}^{\varepsilon},\boldsymbol{\eta}-\tilde{\boldsymbol{\zeta}}_{h}^{\varepsilon})\ge \ell(\boldsymbol{\eta}-\tilde{\boldsymbol{\zeta}}_{h}^{\varepsilon})\quad\text{ for all }\boldsymbol{\eta}\in \boldsymbol{V}_{h}(\omega). $$

The aim of the next lemma, whose formulation is inspired by Lemma 3.1 of [7], is to prove that the uniform boundedness of the family $(\tilde {\boldsymbol {\zeta }}_{h}^{\varepsilon })_{h>0}$, where $\tilde {\boldsymbol {\zeta }}_{h}^{\varepsilon }$ denotes the solution to Problem $\mathcal {P}_{h}^{\varepsilon }(\omega )$. The argument resorts on Cauchy-Schwarz’s inequality, Poincaré-Friedrichs’s inequality (Theorems 6.5-2 and 6.8-1 of [19]), and Young’s inequality (see [47]).

### **Lemma 7**

There exists a constant *C* > 0 such that

49 $$  \|\tilde{\boldsymbol{\zeta}}_{h}^{\varepsilon}\|_{\boldsymbol{V}(\omega)} \le C\quad \text{ for all } h>0. $$

### *Proof*

Fix *h* > 0. Since $K_{3}^{\varepsilon }(\omega ) \subset \tilde {K}_{3,h}^{\varepsilon }(\omega )$, we infer that $J(\tilde {\boldsymbol {\zeta }}_{h}^{\varepsilon }) \le J(\boldsymbol {\zeta }^{\varepsilon })$, where $\tilde {\boldsymbol {\zeta }}_{h}^{\varepsilon }$ and ***ζ***^*ε*^ are respectively the solutions to Problem $\mathcal {P}_{h}^{\varepsilon }(\omega )$ and Problem $\mathcal {P}^{\varepsilon }(\omega )$. Using Cauchy-Schwarz’s inequality, Poincaré-Friedrichs’s inequality, and Young’s inequality, we obtain

$$ \begin{array}{@{}rcl@{}} \frac{\alpha}{2}\|\tilde{\boldsymbol{\zeta}}_{h}^{\varepsilon}\|_{\boldsymbol{V}(\omega)}^{2}&\le& \frac{1}{2}b(\tilde{\boldsymbol{\zeta}}_{h}^{\varepsilon},\tilde{\boldsymbol{\zeta}}_{h}^{\varepsilon})=J(\tilde{\boldsymbol{\zeta}}_{h}^{\varepsilon})+\ell(\tilde{\boldsymbol{\zeta}}_{h}^{\varepsilon}) \le J(\tilde{\boldsymbol{\zeta}}_{h}^{\varepsilon})+C_{\ell} |\tilde{\boldsymbol{\zeta}}_{h}^{\varepsilon}|_{\boldsymbol{V}(\omega)}\\ &\le& J(\tilde{\boldsymbol{\zeta}}_{h}^{\varepsilon})+ \frac{1}{\alpha}C_{\ell}^{2} +\frac{\alpha}{4}|\tilde{\boldsymbol{\zeta}}_{h}^{\varepsilon}|_{\boldsymbol{V}(\omega)}^{2}, \end{array} $$

which in turn implies that

$$ \|\tilde{\boldsymbol{\zeta}}_{h}^{\varepsilon}\|_{\boldsymbol{V}(\omega)}^{2} \le \frac{4}{\alpha}\left( J(\tilde{\boldsymbol{\zeta}}_{h}^{\varepsilon})+ \frac{C_{\ell}^{2}}{\alpha}\right), $$

from which the estimate (49) immediately follows. □

The purpose of the following lemmas, whose formulations are respectively inspired by those of Lemmas 3.2-3.4 of [7], is to estimate the distance between $\tilde {\boldsymbol {\zeta }}_{h}^{\varepsilon }$ and $\boldsymbol {\zeta }^{\varepsilon }\in \boldsymbol {V}_{H}(\omega )\times K_{3}^{\varepsilon }(\omega )$. In what follows, the symbol $\rightharpoonup $ denotes weak convergences as *h* → 0. Strong convergences in the space $\mathcal {C}^{0}(\overline {\omega })$ are meant with respect to the $\sup $-norm.

### **Lemma 8**

The following convergences take place

50 $$ \begin{array}{@{}rcl@{}} \tilde{\boldsymbol{\zeta}}_{h}^{\varepsilon} &\rightharpoonup \boldsymbol{\zeta}^{\varepsilon}\quad\text{ in }\boldsymbol{V}(\omega), \end{array} $$

51 $$ \begin{array}{@{}rcl@{}} \tilde{\zeta}_{3,h}^{\varepsilon} &\to \zeta_{3}^{\varepsilon}\quad\text{ in } \mathcal{C}^{0}(\overline{\omega}). \end{array} $$

### *Proof*

The uniform boundedness of $(\tilde {\boldsymbol {\zeta }}_{h}^{\varepsilon })_{h>0}$ proved in Lemma 7 yields the existence of an element ***ζ***^∗^ ∈***V***(*ω*) such that, up to passing to a subsequence, still denoted $(\tilde {\boldsymbol {\zeta }}_{h}^{\varepsilon })_{h>0}$

$$ \tilde{\boldsymbol{\zeta}}_{h}^{\varepsilon} \rightharpoonup \boldsymbol{\zeta}^{\ast}\quad\text{ in }\boldsymbol{V}(\omega). $$

The functional *J* is clearly sequentially weakly lower semi-continuous. Hence,

$$ J(\boldsymbol{\zeta}^{\ast}) \le \liminf\limits_{h \to 0}J(\tilde{\boldsymbol{\zeta}}_{h}^{\varepsilon}) \le J(\boldsymbol{\zeta}^{\varepsilon}), $$

where the latter inequality is derived in Lemma 7. By the Rellich-Kondrachov theorem, we infer that $\zeta _{3}^{\ast } \in \mathcal {C}^{0}(\overline {\omega })$ and that

$$ \tilde{\zeta}_{3,h}^{\varepsilon} \to \zeta_{3}^{\ast}\quad\text{ in } \mathcal{C}^{0}(\overline{\omega}). $$

It remains to prove ***ζ***^∗^ = ***ζ***^*ε*^. To this end, by the uniqueness of the solution to Problem $\mathcal {P}^{\varepsilon }(\omega )$, it suffices to show that $\zeta _{3}^{\ast } \in K_{3}^{\varepsilon }(\omega )$. Since $\tilde {\zeta }_{3,h}^{\varepsilon }\in \tilde {K}_{3,h}^{\varepsilon }(\omega )$, then

$$ \theta^{\varepsilon}(p)+\tilde{\zeta}_{3,h}^{\varepsilon}(p)\ge 0\quad\text{ for all }p\in \mathcal{V}_{h}. $$

Besides, the following density with respect to the Euclidean norm

$$ \overline{\bigcup\limits_{h>0} \mathcal{V}_{h}}=\overline{\omega}, $$

yields, in conjunction with the previous inequality, that

$$ \theta(q)+\zeta_{3}^{\ast}(q)=\underset{q_{k} \in \mathcal{V}_{h_{k}}}{\lim\limits_{k\to\infty}} \left( \theta(q_{k})+\zeta_{3}^{\ast}(q_{k})\right) =\underset{q_{k} \in \mathcal{V}_{h_{k}}}{\lim\limits_{k\to\infty}} \lim\limits_{h \to 0} \left( \theta(q_{k})+\tilde{\zeta}_{3,h}(q_{k})\right)\ge0, $$

for all $q\in \overline {\omega }$. We have thus shown that $\zeta _{3}^{\ast } \in K_{3}^{\varepsilon }(\omega )$. The convergences (50) and (51) immediately follow. □

Let us denote ${\mathcal{C}}$ the *contact zone* for Problem $\mathcal {P}^{\varepsilon }(\omega )$, i.e.,

$$ \mathcal{C}:=\{y \in \overline{\omega}; \theta^{\varepsilon}(y)+\zeta_{3}^{\varepsilon}(y) =0\}. $$

The set ${\mathcal{C}}$ is compact in $\overline {\omega }$. Since the transverse component of ***ζ***^*ε*^, solution to Problem $\mathcal {P}^{\varepsilon }(\omega )$, belongs to the space ${H^{2}_{0}}(\omega )$ and since *𝜃*^*ε*^ > 0 in $\overline {\omega }$, it follows that ${\mathcal{C}} \cap \gamma =\emptyset $. For any *ρ* > 0, define the set

$$ \mathcal{C}_{\rho}:=\{y \in \overline{\omega}; \text{dist}(y,\mathcal{C})\le \rho\}, $$

where $\text {dist}(y,{\mathcal{C}})$ denotes the distance of any point $y \in \overline {\omega }$ from the set ${\mathcal{C}}$, i.e.,

$$ \text{dist}(y,\mathcal{C}):=\min\limits_{x \in \mathcal{C}}|y-x|. $$

The set ${\mathcal{C}}_{\rho }$ is compact and such that, for sufficiently small *ρ*, ${\mathcal{C}}_{\rho }\cap \gamma =\emptyset $. Moreover, we can choose *ρ* sufficiently small so that ${\mathcal{C}}_{2\rho }\cap \gamma =\emptyset $.

### **Lemma 9**

There exist positive numbers *h*_0_ and *β*_1_ such that

$$ \theta^{\varepsilon}(y)+\tilde{\zeta}_{3,h}^{\varepsilon}(y)\ge \beta_{1}\quad\text{ if } y\in \overline{\omega} \text{ and }\text{dist}(y,\mathcal{C})\ge\rho, $$

for all *h* ≤ *h*_0_.

### *Proof*

Since $(\theta ^{\varepsilon }+\zeta _{3}^{\varepsilon })>0$ outside the contact zone, then it is a fortiori > 0 in the compact set $\{y\in \overline {\omega };\text {dist}(y,{\mathcal{C}})\ge \rho \}$. By virtue of (51), we immediately infer that

$$ (\theta^{\varepsilon}+\tilde{\zeta}_{3,h}^{\varepsilon}) \to (\theta^{\varepsilon}+\zeta_{3}^{\varepsilon})\quad\text{ in }\mathcal{C}^{0}(\overline{\omega}). $$

As a result, there exists *h*_0_ > 0 such that

$$ \theta^{\varepsilon}+\tilde{\zeta}_{3,h}^{\varepsilon}>0\quad\text{ in } \{y\in\overline{\omega};\text{dist}(y,\mathcal{C})\ge\rho\}, $$

and there thus exists *β*_1_ > 0 such that

$$ \theta^{\varepsilon}+\tilde{\zeta}_{3,h}^{\varepsilon}\ge \beta_{1}\quad\text{ in } \{y\in\overline{\omega};\text{dist}(y,\mathcal{C})\ge\rho\}, $$

for all *h* ≤ *h*_0_. □

Following the ideas of [7], we introduce the nodal interpolation operator for the conforming $\mathbb {P}_{1}$ finite element associated with the triangulation $\mathcal {T}_{h}$ and we denote it by $\mathcal {I}_{h}$. By definition of $\mathcal {I}_{h}$, it follows that $\tilde {\zeta }_{3,h}^{\varepsilon }$ and $\mathcal {I}_{h} \tilde {\zeta }_{3,h}^{\varepsilon }$ agree at the vertices of the conforming $\mathbb {P}_{1}$ finite elements. By the linearity of $\mathcal {I}_{h}$ we get

52 $$  \mathcal{I}_{h} \theta^{\varepsilon} +\mathcal{I}_{h} \tilde{\zeta}_{3,h}^{\varepsilon}\ge 0\quad\text{ in }\overline{\omega}, $$

since, again by the properties of $\mathcal {I}_{h}$, the functions $\mathcal {I}_{h} \theta ^{\varepsilon }$ and $\mathcal {I}_{h} \tilde {\zeta }_{3,h}^{\varepsilon }$ are affine over $\overline {\omega }$. By standard interpolation estimates (Theorem 3.1.5 of [14] with *m* = 0, *p* = 2, $q=\infty $, and *k* = 1), we infer

53 $$  \|\eta-\mathcal{I}_{h}\eta\|_{0, \infty,\omega} \le C h |\eta|_{2,\omega}\quad\text{ for all }\eta\in H^{2}(\omega). $$

An application of (53) and (49) yields

54 $$  \|\tilde{\zeta}_{3,h}^{\varepsilon}-\mathcal{I}_{h}\tilde{\zeta}_{3,h}^{\varepsilon}\|_{0, \infty,\omega} \le C h. $$

Since $\theta ^{\varepsilon } \in \mathcal {C}^{3}(\overline {\omega })$, we deduce, by Taylor’s theorem with integral remainder that there exists a positive constant *C* such that

55 $$  \sup\limits_{y \in \overline{\omega}} |\theta^{\varepsilon}-\mathcal{I}_{h}\theta^{\varepsilon}|\le C h^{2}, $$

and such an estimate a fortiori holds for the norm $\|\cdot \|_{L^{\infty }(\omega )}$.

Define

$$ \delta_{h}:=\|(\tilde{\zeta}_{3,h}^{\varepsilon}-\mathcal{I}_{h}\tilde{\zeta}_{3,h}^{\varepsilon})+(\theta^{\varepsilon}-\mathcal{I}_{h}\theta^{\varepsilon})\|_{0, \infty,\omega}. $$

In view of (54) and (55), it is straightforward to verify that there exists a positive constant *C* such that

56 $$  \delta_{h}\le Ch. $$

The proof of the next result is obtained by Lemmas 7–9.

### **Lemma 10**

There exists a positive constant *C* such that

57 $$  |\boldsymbol{\zeta}^{\varepsilon}-\tilde{\boldsymbol{\zeta}}_{h}^{\varepsilon}|_{\boldsymbol{V}(\omega)} \le Ch. $$

### *Proof*

Let *h*_0_ and *β*_1_ be as in Lemma 9 and let us assume, without loss of generality, that *h* ≤ *h*_0_. By the property (52) of the nodal interpolation operator $\mathcal {I}_{h}$, we have

$$ \theta^{\varepsilon}+\tilde{\zeta}_{3,h}^{\varepsilon} \ge \theta^{\varepsilon}+\tilde{\zeta}_{3,h}^{\varepsilon}-\mathcal{I}_{h}\theta^{\varepsilon}-\mathcal{I}_{h}\tilde{\zeta}_{3,h}^{\varepsilon} \ge -\delta_{h}\quad\text{ in } \mathcal{C}_{\rho}. $$

By virtue of (56), it is also licit to assume *δ*_*h*_ < *β*. Let *f* be a continuous function defined over *ω* as follows

58 $$ \begin{array}{@{}rcl@{}} f&=&1\quad\text{ in }\mathcal{C}_{\rho}, \end{array} $$

59 $$ \begin{array}{@{}rcl@{}} f&=&0\quad\text{ in }\omega\setminus \mathcal{C}_{2\rho}. \end{array} $$

Let *ϱ* denote a mollifier whose support is a subset of ${\mathcal{C}}_{2\rho }$. Define the function $\varphi \in \mathcal D(\omega )$ by

$$ \varphi:=\varrho \ast f. $$

It follows that

60 $$ \begin{array}{@{}rcl@{}} 0\le\varphi&\le& 1 \text{ in }\omega, \end{array} $$

61 $$ \begin{array}{@{}rcl@{}} \varphi&=&1 \text{ in }\mathcal{C}_{\rho}, \end{array} $$

62 $$ \begin{array}{@{}rcl@{}} \varphi&=&0 \text{ in }\omega\setminus \mathcal{C}_{2\rho}. \end{array} $$

We claim that the function $\hat {\zeta }_{3,h}^{\varepsilon }:=\tilde {\zeta }_{3,h}^{\varepsilon }+\delta _{h}\varphi $ belongs to the set $K_{3}^{\varepsilon }(\omega )$. It is straightforward to verify that $\hat {\zeta }_{3,h}^{\varepsilon }=\partial _{\nu }\hat {\zeta }_{3,h}^{\varepsilon }=0$ on *γ*. It thus remains to show that $(\theta ^{\varepsilon }+\hat {\zeta }_{3,h}^{\varepsilon })\ge 0$ in $\overline {\omega }$. To this aim, we will distinguish three cases:

### *Case 1* ($x \in \omega \setminus {\mathcal{C}}_{2\rho }$)

In this case, by virtue of (62), we get $\tilde {\zeta }_{3,h}^{\varepsilon }=\hat {\zeta }_{3,h}^{\varepsilon }$ and the conclusion immediately follows by Lemma 9.

### *Case 2* ($x \in {\mathcal{C}}_{2\rho } \setminus {\mathcal{C}}_{\rho }$)

In this case, by virtue of (60) and Lemma 9, we get

$$ \theta^{\varepsilon}+\hat{\zeta}_{3,h}^{\varepsilon}=\theta^{\varepsilon}+\tilde{\zeta}_{3,h}^{\varepsilon}+\delta_{h} \varphi \ge \beta_{1}>0. $$

### *Case 3* ($x \in {\mathcal{C}}_{\rho }$)

In this case, by virtue of (61), we get

$$ \theta^{\varepsilon}+\hat{\zeta}_{3,h}^{\varepsilon}=\theta^{\varepsilon}+\tilde{\zeta}_{3,h}^{\varepsilon}+\delta_{h} \ge 0. $$

In conclusion, we have shown that $\hat {\zeta }_{3,h}^{\varepsilon }$ belongs to the set $K_{3}^{\varepsilon }(\omega )$. An application of (49) gives

$$ \begin{array}{@{}rcl@{}} J\left( (\tilde{\zeta}_{1,h}^{\varepsilon}, \tilde{\zeta}_{2,h}^{\varepsilon}, \hat{\zeta}_{3,h}^{\varepsilon})\right)&=& \frac{1}{2}b\left( \tilde{\boldsymbol{\zeta}}_{h}^{\varepsilon}+(0,0,\delta_{h} \varphi),\tilde{\boldsymbol{\zeta}}_{h}^{\varepsilon}+(0,0,\delta_{h} \varphi)\right)-\ell\left( \tilde{\boldsymbol{\zeta}}_{h}^{\varepsilon}+(0,0,\delta_{h} \varphi)\right)\\ &=&\left[\frac{1}{2}b(\tilde{\boldsymbol{\zeta}}_{h}^{\varepsilon},\tilde{\boldsymbol{\zeta}}_{h}^{\varepsilon})-\ell(\tilde{\boldsymbol{\zeta}}_{h}^{\varepsilon})\right] + b\left( \tilde{\boldsymbol{\zeta}}_{h}^{\varepsilon},(0,0,\delta_{h} \varphi)\right)\\ &&+\frac{1}{2}b\left( (0,0,\delta_{h} \varphi),(0,0,\delta_{h} \varphi)\right)-\ell\left( (0,0,\delta_{h} \varphi)\right)\\ &=&J(\tilde{\boldsymbol{\zeta}}_{h}^{\varepsilon})+b\left( \tilde{\boldsymbol{\zeta}}_{h}^{\varepsilon},(0,0,\delta_{h} \varphi)\right)-\ell\left( (0,0,\delta_{h} \varphi)\right)\\ &&+\frac{1}{2}b\left( (0,0,\delta_{h} \varphi), (0,0,\delta_{h} \varphi)\right) \le J(\tilde{\boldsymbol{\zeta}}_{h}^{\varepsilon}) +C \delta_{h}. \end{array} $$

By the ***V***(*ω*)-ellipticity of *b*(⋅,⋅) (cf. Theorem 3.6-1 of [16]), the intermediary inequality (48), and the fact that $\tilde {\zeta }_{3,h}^{\varepsilon }$ is in $K_{3}^{\varepsilon }(\omega )$ (see Lemma 10), we obtain

$$ \begin{array}{@{}rcl@{}} \frac{\alpha}{2} \|\boldsymbol{\zeta}^{\varepsilon}-\tilde{\boldsymbol{\zeta}}_{h}^{\varepsilon}\|_{\boldsymbol{V}(\omega)} &\le& \frac{1}{2}b(\boldsymbol{\zeta}^{\varepsilon}-\tilde{\boldsymbol{\zeta}}_{h}^{\varepsilon}, \boldsymbol{\zeta}^{\varepsilon}-\tilde{\boldsymbol{\zeta}}_{h}^{\varepsilon})\\ &=&\frac{1}{2}b(\boldsymbol{\zeta}^{\varepsilon},\boldsymbol{\zeta}^{\varepsilon})-b(\tilde{\boldsymbol{\zeta}}_{h}^{\varepsilon}, \boldsymbol{\zeta}^{\varepsilon}-\tilde{\boldsymbol{\zeta}}_{h}^{\varepsilon})-\frac{1}{2} b(\tilde{\boldsymbol{\zeta}}_{h}^{\varepsilon},\tilde{\boldsymbol{\zeta}}_{h}^{\varepsilon})\\ &\le& \frac{1}{2}b(\boldsymbol{\zeta}^{\varepsilon},\boldsymbol{\zeta}^{\varepsilon})-\ell(\boldsymbol{\zeta}^{\varepsilon}-\tilde{\boldsymbol{\zeta}}_{h}^{\varepsilon})-\frac{1}{2}b(\tilde{\boldsymbol{\zeta}}_{h}^{\varepsilon},\tilde{\boldsymbol{\zeta}}_{h}^{\varepsilon})\\ &=&J(\boldsymbol{\zeta}^{\varepsilon})-J(\tilde{\boldsymbol{\zeta}}_{h}^{\varepsilon})\le J\left( (\tilde{\zeta}_{1,h}^{\varepsilon},\tilde{\zeta}_{2,h}^{\varepsilon},\hat{\zeta}_{3,h}^{\varepsilon})\right)-J(\tilde{\boldsymbol{\zeta}}_{h}^{\varepsilon})\\ &\le& C \delta_{h}. \end{array} $$

The conclusion immediately follows by (56). □

## Convergence analysis

The next lemma, inspired by Lemma 4.2 of [7], provides an estimate for the term $[b_{h}(\boldsymbol {\zeta }^{\varepsilon }, \boldsymbol {{\varPi }}_{h}\boldsymbol {\zeta }^{\varepsilon }-\boldsymbol {\zeta }_{h}^{\varepsilon })-\ell (\boldsymbol {{\varPi }}_{h}\boldsymbol {\zeta }^{\varepsilon }-\boldsymbol {\zeta }_{h}^{\varepsilon })]$, which thus allows us to complete the error analysis. The proof relies on Lemma 5, Lemma 10, and standard interpolation estimates (see, e.g., [14]).

### **Lemma 11**

There exists a positive constant *C* such that

63 $$  b_{h}(\boldsymbol{\zeta}^{\varepsilon}, \boldsymbol{{\varPi}}_{h}\boldsymbol{\zeta}^{\varepsilon}-\boldsymbol{\zeta}_{h}^{\varepsilon})-\ell(\boldsymbol{{\varPi}}_{h}\boldsymbol{\zeta}^{\varepsilon}-\boldsymbol{\zeta}_{h}^{\varepsilon}) \le C \sqrt{h}\left( \sqrt{h}+\|\boldsymbol{{\varPi}}_{h} \boldsymbol{\zeta}^{\varepsilon}-\boldsymbol{\zeta}_{h}^{\varepsilon}\|\right). $$

### *Proof*

Observe that we can write

$$ \begin{array}{@{}rcl@{}} b_{h}(\boldsymbol{\zeta}^{\varepsilon}, \boldsymbol{{\varPi}}_{h}\boldsymbol{\zeta}^{\varepsilon}-\boldsymbol{\zeta}_{h}^{\varepsilon})&=&b_{h}(\boldsymbol{\zeta}^{\varepsilon}, \tilde{\boldsymbol{E}}_{h}\boldsymbol{{\varPi}}_{h}\boldsymbol{\zeta}^{\varepsilon}-\tilde{\boldsymbol{E}}_{h}\boldsymbol{\zeta}_{h}^{\varepsilon}) +b_{h}(\boldsymbol{\zeta}^{\varepsilon},\boldsymbol{{\varPi}}_{h}\boldsymbol{\zeta}^{\varepsilon}-\boldsymbol{\zeta}_{h}^{\varepsilon}-\tilde{\boldsymbol{E}}_{h}(\boldsymbol{{\varPi}}_{h}\boldsymbol{\zeta}^{\varepsilon}-\boldsymbol{\zeta}_{h}^{\varepsilon}))\\ &=&b(\boldsymbol{\zeta}^{\varepsilon},\tilde{\boldsymbol{E}}_{h}(\boldsymbol{{\varPi}}_{h}\boldsymbol{\zeta}^{\varepsilon}-\boldsymbol{\zeta}_{h}^{\varepsilon})) +b_{h}(\boldsymbol{\zeta}^{\varepsilon},\boldsymbol{{\varPi}}_{h}\boldsymbol{\zeta}^{\varepsilon}-\boldsymbol{\zeta}_{h}^{\varepsilon}-\tilde{\boldsymbol{E}}_{h}(\boldsymbol{{\varPi}}_{h}\boldsymbol{\zeta}^{\varepsilon}-\boldsymbol{\zeta}_{h}^{\varepsilon}))\\ &\le& b(\boldsymbol{\zeta}^{\varepsilon},\tilde{\boldsymbol{E}}_{h}(\boldsymbol{{\varPi}}_{h}\boldsymbol{\zeta}^{\varepsilon}-\boldsymbol{\zeta}_{h}^{\varepsilon})) + C h \|\boldsymbol{\zeta}^{\varepsilon}\|_{\omega} \|\boldsymbol{{\varPi}}_{h}\boldsymbol{\zeta}^{\varepsilon}-\boldsymbol{\zeta}_{h}^{\varepsilon}\|, \end{array} $$

where the latter inequality holds by (39). We have thus shown that there exists a constant *C* > 0 such that

64 $$  b_{h}(\boldsymbol{\zeta}^{\varepsilon}, \boldsymbol{{\varPi}}_{h}\boldsymbol{\zeta}^{\varepsilon}-\boldsymbol{\zeta}_{h}^{\varepsilon}) \le b(\boldsymbol{\zeta}^{\varepsilon},\tilde{\boldsymbol{E}}_{h}(\boldsymbol{{\varPi}}_{h}\boldsymbol{\zeta}^{\varepsilon}-\boldsymbol{\zeta}_{h}^{\varepsilon}))+ C h \|\boldsymbol{\zeta}^{\varepsilon}\|_{\omega} \|\boldsymbol{{\varPi}}_{h}\boldsymbol{\zeta}^{\varepsilon}-\boldsymbol{\zeta}_{h}^{\varepsilon}\|. $$

Let us now estimate the term $b(\boldsymbol {\zeta }^{\varepsilon },\tilde {\boldsymbol {E}}_{h}(\boldsymbol {{\varPi }}_{h}\boldsymbol {\zeta }^{\varepsilon }-\boldsymbol {\zeta }_{h}^{\varepsilon }))$. We first notice that we can write it in the more suitable equivalent form

65 $$  b(\boldsymbol{\zeta}^{\varepsilon},\tilde{\boldsymbol{E}}_{h}(\boldsymbol{{\varPi}}_{h}\boldsymbol{\zeta}^{\varepsilon}-\boldsymbol{\zeta}_{h}^{\varepsilon}))=b(\tilde{\boldsymbol{\zeta}}_{h}^{\varepsilon},\tilde{\boldsymbol{E}}_{h}(\boldsymbol{{\varPi}}_{h}\boldsymbol{\zeta}^{\varepsilon}-\boldsymbol{\zeta}_{h}^{\varepsilon}))+b(\boldsymbol{\zeta}^{\varepsilon}-\tilde{\boldsymbol{\zeta}}_{h}^{\varepsilon},\tilde{\boldsymbol{E}}_{h}(\boldsymbol{{\varPi}}_{h}\boldsymbol{\zeta}^{\varepsilon}-\boldsymbol{\zeta}_{h}^{\varepsilon})). $$

Using (38), (57), the continuity of *b*(⋅,⋅), and the Poincaré-Friedrichs inequality (Theorems 6.5-2 and 6.8-1 of [19]), we can estimate the second term in the right-hand side of (65) as follows

$$ b(\boldsymbol{\zeta}^{\varepsilon}-\tilde{\boldsymbol{\zeta}}_{h}^{\varepsilon},\tilde{\boldsymbol{E}}_{h}(\boldsymbol{{\varPi}}_{h}\boldsymbol{\zeta}^{\varepsilon}-\boldsymbol{\zeta}_{h}^{\varepsilon}))\le C |\boldsymbol{\zeta}^{\varepsilon}-\tilde{\boldsymbol{\zeta}}_{h}^{\varepsilon}|_{\boldsymbol{V}(\omega)} |\tilde{\boldsymbol{E}}_{h}(\boldsymbol{{\varPi}}_{h}\boldsymbol{\zeta}^{\varepsilon}-\boldsymbol{\zeta}_{h}^{\varepsilon})|_{\boldsymbol{V}(\omega)} \le C \sqrt{h}\|\boldsymbol{{\varPi}}_{h}\boldsymbol{\zeta}^{\varepsilon}-\boldsymbol{\zeta}_{h}^{\varepsilon}\|. $$

As a result, we obtain

66 $$  b(\boldsymbol{\zeta}^{\varepsilon}-\tilde{\boldsymbol{\zeta}}_{h}^{\varepsilon},\tilde{\boldsymbol{E}}_{h}(\boldsymbol{{\varPi}}_{h}\boldsymbol{\zeta}^{\varepsilon}-\boldsymbol{\zeta}_{h}^{\varepsilon}))\le C \sqrt{h}\|\boldsymbol{{\varPi}}_{h}\boldsymbol{\zeta}^{\varepsilon}-\boldsymbol{\zeta}_{h}^{\varepsilon}\|. $$

Regarding the first term in the right-hand side of (65), we observe that (48) yields

67 $$  \begin{array}{llll} b(\tilde{\boldsymbol{\zeta}}_{h}^{\varepsilon},\tilde{\boldsymbol{E}}_{h}(\boldsymbol{{\varPi}}_{h}\boldsymbol{\zeta}^{\varepsilon}-\boldsymbol{\zeta}_{h}^{\varepsilon}))&= b(\tilde{\boldsymbol{\zeta}}_{h}^{\varepsilon},\tilde{\boldsymbol{\zeta}}_{h}^{\varepsilon}-\tilde{\boldsymbol{E}}_{h}\boldsymbol{\zeta}_{h}^{\varepsilon})+b(\tilde{\boldsymbol{\zeta}}_{h}^{\varepsilon},\tilde{\boldsymbol{E}}_{h}\boldsymbol{{\varPi}}_{h}\boldsymbol{\zeta}^{\varepsilon}-\boldsymbol{\zeta}_{h}^{\varepsilon})\\ &\le \ell(\tilde{\boldsymbol{\zeta}}_{h}^{\varepsilon}-\tilde{\boldsymbol{E}}_{h} \boldsymbol{\zeta}_{h}^{\varepsilon})+b(\tilde{\boldsymbol{\zeta}}_{h}^{\varepsilon}, \tilde{\boldsymbol{E}}_{h} \boldsymbol{{\varPi}}_{h} \boldsymbol{\zeta}^{\varepsilon}-\tilde{\boldsymbol{\zeta}}_{h}^{\varepsilon}). \end{array} $$

We note that

68 $$ \begin{array}{@{}rcl@{}} b(\tilde{\boldsymbol{\zeta}}_{h}^{\varepsilon}, \tilde{\boldsymbol{E}}_{h} \boldsymbol{{\varPi}}_{h} \boldsymbol{\zeta}^{\varepsilon}-\tilde{\boldsymbol{\zeta}}_{h}^{\varepsilon})&=& b(\tilde{\boldsymbol{\zeta}}_{h}^{\varepsilon}-\boldsymbol{\zeta}^{\varepsilon}, \tilde{\boldsymbol{E}}_{h} \boldsymbol{{\varPi}}_{h} \boldsymbol{\zeta}^{\varepsilon}-\tilde{\boldsymbol{\zeta}}_{h}^{\varepsilon}) +b(\boldsymbol{\zeta}^{\varepsilon}, \tilde{\boldsymbol{E}}_{h} \boldsymbol{{\varPi}}_{h} \boldsymbol{\zeta}^{\varepsilon}-\boldsymbol{\zeta}^{\varepsilon})\\ &&+b(\boldsymbol{\zeta}^{\varepsilon},\boldsymbol{\zeta}^{\varepsilon}-\tilde{\boldsymbol{\zeta}}_{h}^{\varepsilon}). \end{array} $$

We estimate the sum of the first two terms of the right-hand side of (68) as follows

69 $$ \begin{array}{@{}rcl@{}} &&|b(\tilde{\boldsymbol{\zeta}}_{h}^{\varepsilon}-\boldsymbol{\zeta}^{\varepsilon}, \tilde{\boldsymbol{E}}_{h} \boldsymbol{{\varPi}}_{h} \boldsymbol{\zeta}^{\varepsilon}-\tilde{\boldsymbol{\zeta}}_{h}^{\varepsilon}) +b(\boldsymbol{\zeta}^{\varepsilon}, \tilde{\boldsymbol{E}}_{h} \boldsymbol{{\varPi}}_{h} \boldsymbol{\zeta}^{\varepsilon}-\boldsymbol{\zeta}^{\varepsilon})| \\ &&\le C |\boldsymbol{\zeta}^{\varepsilon}-\tilde{\boldsymbol{\zeta}}_{h}^{\varepsilon}|_{\boldsymbol{V}(\omega)} |(\tilde{\boldsymbol{\zeta}}_{h}^{\varepsilon}-\boldsymbol{\zeta}^{\varepsilon})+(\boldsymbol{\zeta}^{\varepsilon}-\tilde{\boldsymbol{E}}_{h}\boldsymbol{{\varPi}}_{h}\boldsymbol{\zeta}^{\varepsilon})|_{\boldsymbol{V}(\omega)}\\ &&+C \|\boldsymbol{\zeta}^{\varepsilon}\|_{\omega}|\boldsymbol{\zeta}^{\varepsilon}-\tilde{\boldsymbol{E}}_{h}\boldsymbol{{\varPi}}_{h} \boldsymbol{\zeta}^{\varepsilon}|_{\boldsymbol{V}(\omega)}\\ &&\le C \left( |\boldsymbol{\zeta}^{\varepsilon}-\tilde{\boldsymbol{\zeta}}_{h}^{\varepsilon}|_{\boldsymbol{V}(\omega)}^{2} + |\boldsymbol{\zeta}^{\varepsilon}-\tilde{\boldsymbol{\zeta}}_{h}^{\varepsilon}|_{\boldsymbol{V}(\omega)}|\boldsymbol{\zeta}^{\varepsilon}-\tilde{\boldsymbol{E}}_{h}\boldsymbol{{\varPi}}_{h} \boldsymbol{\zeta}^{\varepsilon}|_{\boldsymbol{V}(\omega)}\right.\\ &&\left.+\|\boldsymbol{\zeta}^{\varepsilon}\|_{\omega}|\boldsymbol{\zeta}^{\varepsilon}-\tilde{\boldsymbol{E}}_{h}\boldsymbol{{\varPi}}_{h} \boldsymbol{\zeta}^{\varepsilon}|_{\boldsymbol{V}(\omega)}\right)\le C h, \end{array} $$

where the latter inequality is obtained by virtue of (36) (for the transverse component only), the Poincaré-Friedrichs inequality (Theorems 6.5-2 and 6.8-1 of [19]), and standard interpolation estimates (Theorem 3.1.5 of [14] with *m* = *k* = 1 and *p* = *q* = 2). Let us assume, without loss of generality, that *h* is sufficiently small so that the definition of $\hat {\zeta }_{3,h}^{\varepsilon }$ is justified (see Lemma 10).

An application of (10), (36) (for the transverse component only), and standard interpolation estimates (Theorem 3.1.5 of [14] with *m* = 0, *k* = 1, and *p* = *q* = 2) yields

$$ \begin{array}{@{}rcl@{}} b(\boldsymbol{\zeta}^{\varepsilon},\boldsymbol{\zeta}^{\varepsilon}-\tilde{\boldsymbol{\zeta}}_{h}^{\varepsilon})&=&b\left( \boldsymbol{\zeta}^{\varepsilon},\boldsymbol{\zeta}^{\varepsilon}-(\tilde{\zeta}_{1,h}^{\varepsilon},\tilde{\zeta}_{2,h}^{\varepsilon},\hat{\zeta}_{3,h}^{\varepsilon})\right)+b\left( \boldsymbol{\zeta}^{\varepsilon},(0,0,\delta_{h} \varphi)\right)\\ &\le& \ell\left( \boldsymbol{\zeta}^{\varepsilon}-(\tilde{\zeta}_{1,h}^{\varepsilon},\tilde{\zeta}_{2,h}^{\varepsilon},\hat{\zeta}_{3,h}^{\varepsilon})\right) +\delta_{h} b\left( \boldsymbol{\zeta}^{\varepsilon},(0,0, \varphi)\right)\\ &=&\ell(\tilde{\boldsymbol{E}}_{h}\boldsymbol{{\varPi}}_{h}\boldsymbol{\zeta}^{\varepsilon}-\tilde{\boldsymbol{\zeta}}_{h}^{\varepsilon})+\ell(\boldsymbol{\zeta}^{\varepsilon}-\tilde{\boldsymbol{E}}_{h}\boldsymbol{{\varPi}}_{h}\boldsymbol{\zeta}^{\varepsilon})\\ &&-\delta_{h}\left[\ell\left( (0,0,\varphi)\right)-b\left( \boldsymbol{\zeta}^{\varepsilon},(0,0,\varphi)\right)\right]\\ &\le& \ell(\tilde{\boldsymbol{E}}_{h}\boldsymbol{{\varPi}}_{h}\boldsymbol{\zeta}^{\varepsilon}-\tilde{\boldsymbol{\zeta}}_{h}^{\varepsilon})+ C h \|\boldsymbol{\zeta}^{\varepsilon}\|_{\omega}\\ &&-\delta_{h}\left[\ell\left( (0,0,\varphi)\right)-b\left( \boldsymbol{\zeta}^{\varepsilon},(0,0,\varphi)\right)\right]\\ &\le& \ell(\tilde{\boldsymbol{E}}_{h}\boldsymbol{{\varPi}}_{h}\boldsymbol{\zeta}^{\varepsilon}-\tilde{\boldsymbol{\zeta}}_{h}^{\varepsilon})+ C h. \end{array} $$

In conclusion, we have shown that there exists a constant *C* > 0 such that

70 $$  b(\boldsymbol{\zeta}^{\varepsilon},\boldsymbol{\zeta}^{\varepsilon}-\tilde{\boldsymbol{\zeta}}_{h}^{\varepsilon}) \le \ell(\tilde{\boldsymbol{E}}_{h}\boldsymbol{{\varPi}}_{h}\boldsymbol{\zeta}^{\varepsilon}-\tilde{\boldsymbol{\zeta}}_{h}^{\varepsilon})+ Ch. $$

An application of (64)–(70), Hölder’s inequality, and (38) yields

$$ \begin{array}{@{}rcl@{}} &&b_{h}(\boldsymbol{\zeta}^{\varepsilon},\boldsymbol{{\varPi}}_{h} \boldsymbol{\zeta}^{\varepsilon}-\boldsymbol{\zeta}_{h}^{\varepsilon})-\ell(\boldsymbol{{\varPi}}_{h}\boldsymbol{\zeta}^{\varepsilon}-\boldsymbol{\zeta}_{h}^{\varepsilon}) \le b\left( \boldsymbol{\zeta}^{\varepsilon},\tilde{\boldsymbol{E}}_{h}(\boldsymbol{{\varPi}}_{h}\boldsymbol{\zeta}^{\varepsilon}-\boldsymbol{\zeta}_{h}^{\varepsilon})\right) \\ &&+ C h \|\boldsymbol{\zeta}^{\varepsilon}\|_{\omega} \|\boldsymbol{{\varPi}}_{h}\boldsymbol{\zeta}^{\varepsilon}-\boldsymbol{\zeta}_{h}^{\varepsilon}\|- \ell(\boldsymbol{{\varPi}}_{h}\boldsymbol{\zeta}^{\varepsilon}-\boldsymbol{\zeta}_{h}^{\varepsilon})\\ &&=b\left( \tilde{\boldsymbol{\zeta}}_{h}^{\varepsilon}, \tilde{\boldsymbol{E}}_{h}(\boldsymbol{{\varPi}}_{h}\boldsymbol{\zeta}^{\varepsilon}-\boldsymbol{\zeta}_{h}^{\varepsilon})\right) +b\left( \boldsymbol{\zeta}^{\varepsilon}-\tilde{\boldsymbol{\zeta}}_{h}^{\varepsilon}, \tilde{\boldsymbol{E}}_{h}(\boldsymbol{{\varPi}}_{h}\boldsymbol{\zeta}^{\varepsilon}-\boldsymbol{\zeta}_{h}^{\varepsilon})\right) \\ &&+C h \|\boldsymbol{{\varPi}}_{h}\boldsymbol{\zeta}^{\varepsilon}-\boldsymbol{\zeta}_{h}^{\varepsilon}\|-\ell(\boldsymbol{{\varPi}}_{h}\boldsymbol{\zeta}^{\varepsilon}-\boldsymbol{\zeta}_{h}^{\varepsilon})\\ &&\le b\left( \tilde{\boldsymbol{\zeta}}_{h}^{\varepsilon}, \tilde{\boldsymbol{E}}_{h}(\boldsymbol{{\varPi}}_{h}\boldsymbol{\zeta}^{\varepsilon}-\boldsymbol{\zeta}_{h}^{\varepsilon})\right) + C \sqrt{h}\|\boldsymbol{{\varPi}}_{h}\boldsymbol{\zeta}^{\varepsilon}-\boldsymbol{\zeta}_{h}^{\varepsilon}\| -\ell(\boldsymbol{{\varPi}}_{h}\boldsymbol{\zeta}^{\varepsilon}-\boldsymbol{\zeta}_{h}^{\varepsilon})\\ &&\le \ell(\tilde{\boldsymbol{\zeta}}_{h}^{\varepsilon}-\tilde{\boldsymbol{E}}_{h} \boldsymbol{\zeta}_{h}^{\varepsilon})+b\left( \tilde{\boldsymbol{\zeta}}_{h}^{\varepsilon},\tilde{\boldsymbol{E}}_{h} \boldsymbol{{\varPi}}_{h}\boldsymbol{\zeta}^{\varepsilon}-\tilde{\boldsymbol{\zeta}}_{h}^{\varepsilon}\right) +C \sqrt{h}\|\boldsymbol{{\varPi}}_{h}\boldsymbol{\zeta}^{\varepsilon}-\boldsymbol{\zeta}_{h}^{\varepsilon}\|-\ell(\boldsymbol{{\varPi}}_{h}\boldsymbol{\zeta}^{\varepsilon}-\boldsymbol{\zeta}_{h}^{\varepsilon})\\ &&=\left[b\left( \tilde{\boldsymbol{\zeta}}_{h}^{\varepsilon}-\boldsymbol{\zeta}^{\varepsilon}, \tilde{\boldsymbol{E}}_{h} \boldsymbol{{\varPi}}_{h} \boldsymbol{\zeta}^{\varepsilon}-\tilde{\boldsymbol{\zeta}}_{h}^{\varepsilon}\right)+b\left( \boldsymbol{\zeta}^{\varepsilon},\tilde{\boldsymbol{E}}_{h}\boldsymbol{{\varPi}}_{h} \boldsymbol{\zeta}^{\varepsilon}-\boldsymbol{\zeta}^{\varepsilon}\right)\right]\\ &&+b(\boldsymbol{\zeta}^{\varepsilon},\boldsymbol{\zeta}^{\varepsilon}-\tilde{\boldsymbol{\zeta}}_{h}^{\varepsilon}) +\ell(\tilde{\boldsymbol{\zeta}}_{h}^{\varepsilon}-\tilde{\boldsymbol{E}}_{h} \boldsymbol{\zeta}_{h}^{\varepsilon})\\ && +C \sqrt{h} \|\boldsymbol{{\varPi}}_{h}\boldsymbol{\zeta}^{\varepsilon}-\boldsymbol{\zeta}_{h}^{\varepsilon}\|-\ell(\boldsymbol{{\varPi}}_{h}\boldsymbol{\zeta}^{\varepsilon}-\boldsymbol{\zeta}_{h}^{\varepsilon})\\ &&\le Ch + C \sqrt{h}\|\boldsymbol{{\varPi}}_{h}\boldsymbol{\zeta}^{\varepsilon}-\boldsymbol{\zeta}_{h}^{\varepsilon}\|+b(\boldsymbol{\zeta}^{\varepsilon},\boldsymbol{\zeta}^{\varepsilon}-\tilde{\boldsymbol{\zeta}}_{h}^{\varepsilon})\\ &&+\ell(\tilde{\boldsymbol{\zeta}}_{h}^{\varepsilon}-\tilde{\boldsymbol{E}}_{h} \boldsymbol{\zeta}_{h}^{\varepsilon})-\ell(\boldsymbol{{\varPi}}_{h}\boldsymbol{\zeta}^{\varepsilon}-\boldsymbol{\zeta}_{h}^{\varepsilon})\\ &&\le Ch +C \sqrt{h}\|\boldsymbol{{\varPi}}_{h}\boldsymbol{\zeta}^{\varepsilon}-\boldsymbol{\zeta}_{h}^{\varepsilon}\|-\ell(\boldsymbol{{\varPi}}_{h}\boldsymbol{\zeta}^{\varepsilon}-\boldsymbol{\zeta}_{h}^{\varepsilon})+\ell(\tilde{\boldsymbol{E}}_{h}(\boldsymbol{{\varPi}}_{h}\boldsymbol{\zeta}^{\varepsilon}-\boldsymbol{\zeta}_{h}^{\varepsilon}))\\ &&\le C h + C\sqrt{h}\|\boldsymbol{{\varPi}}_{h}\boldsymbol{\zeta}^{\varepsilon}-\boldsymbol{\zeta}_{h}^{\varepsilon}\|. \end{array} $$

To sum up, we have shown that there exists *C* > 0 such that

$$ b_{h}(\boldsymbol{\zeta}^{\varepsilon},\boldsymbol{{\varPi}}_{h} \boldsymbol{\zeta}^{\varepsilon}-\boldsymbol{\zeta}_{h}^{\varepsilon})-\ell(\boldsymbol{{\varPi}}_{h}\boldsymbol{\zeta}^{\varepsilon}-\boldsymbol{\zeta}_{h}^{\varepsilon})\le C \sqrt{h}(\sqrt{h}+\|\boldsymbol{{\varPi}}_{h}\boldsymbol{\zeta}^{\varepsilon}-\boldsymbol{\zeta}_{h}^{\varepsilon}\|), $$

which completes the proof. □

We are now in a position to recover the error estimate in terms of the norm ∥⋅∥, whose definition is recalled here below:

$$ \|\boldsymbol{\eta}_{h}\|:=\|\eta_{1,h}\|_{1,\omega}+\|\eta_{2,h}\|_{1,\omega}+\|\eta_{3,h}\|_{h}\quad\text{ for all } \boldsymbol{\eta}_{h} \in \tilde{\boldsymbol{V}}_{h}. $$

The proof of the error estimate, which constitutes the main result of this paper, resorts to Lemma 6, Lemma 11, and Young’s inequality (cf. [47]).

### **Theorem 1**

There exists a positive constant *C* such that

71 $$  \|\boldsymbol{\zeta}^{\varepsilon}-\boldsymbol{\zeta}_{h}^{\varepsilon}\|\le C \sqrt{h}. $$

### *Proof*

An application of Lemma 6, Lemma 11, (11), and Young’s inequality yields

$$ \begin{array}{@{}rcl@{}} \|\boldsymbol{\zeta}^{\varepsilon}-\boldsymbol{\zeta}_{h}^{\varepsilon}\|^{2} &\le& C_{1}\|\boldsymbol{{\varPi}}_{h}\boldsymbol{\zeta}^{\varepsilon}-\boldsymbol{\zeta}^{\varepsilon}\|^{2} +C_{2}\left[b(\boldsymbol{\zeta}^{\varepsilon},\boldsymbol{{\varPi}}_{h}\boldsymbol{\zeta}^{\varepsilon}-\boldsymbol{\zeta}_{h}^{\varepsilon})-\ell(\boldsymbol{{\varPi}}_{h}\boldsymbol{\zeta}^{\varepsilon}-\boldsymbol{\zeta}_{h}^{\varepsilon})\right]\\ &\le& C_{1}\|\boldsymbol{{\varPi}}_{h}\boldsymbol{\zeta}^{\varepsilon}-\boldsymbol{\zeta}^{\varepsilon}\|^{2} + C\sqrt{h}(\sqrt{h}+\|\boldsymbol{{\varPi}}_{h}\boldsymbol{\zeta}^{\varepsilon}-\boldsymbol{\zeta}_{h}^{\varepsilon}\|)\\ &\le& C h+ C\sqrt{h}(\sqrt{h}+\|\boldsymbol{{\varPi}}_{h}\boldsymbol{\zeta}^{\varepsilon}-\boldsymbol{\zeta}^{\varepsilon}\|+\|\boldsymbol{\zeta}^{\varepsilon}-\boldsymbol{\zeta}_{h}^{\varepsilon}\|)\\ &\le& C\left( h + \sqrt{h}\|\boldsymbol{\zeta}^{\varepsilon}-\boldsymbol{\zeta}_{h}^{\varepsilon}\|\right)\\ &\le& C\left( h + \frac{C h}{2}+\frac{1}{2C}\|\boldsymbol{\zeta}^{\varepsilon}-\boldsymbol{\zeta}_{h}^{\varepsilon}\|^{2}\right)\le Ch+\frac{1}{2}\|\boldsymbol{\zeta}^{\varepsilon}-\boldsymbol{\zeta}_{h}^{\varepsilon}\|^{2}. \end{array} $$

In conclusion, we obtain

$$ \|\boldsymbol{\zeta}^{\varepsilon}-\boldsymbol{\zeta}_{h}^{\varepsilon}\|^{2} \le Ch, $$

and (71) is thus proved. □
